# Setting the geological scene for the origin of life and continuing open questions about its emergence

**DOI:** 10.3389/fspas.2022.1095701

**Published:** 2023-01-05

**Authors:** Frances Westall, André Brack, Alberto G. Fairén, Mitchell D. Schulte

**Affiliations:** 1 Centre de Biophysique Moléculaire, CNRS, Orléans, France; 2 Centro de Astrobiología (CAB, CSIC-INTA), Madrid, Spain; 3 Cornell University, Ithaca, NY, United States,; 4 NASA Headquarters, Washington, DC, United States

**Keywords:** origin of life, early earth, prebiotic environments, volcanic rocks, stochastic chemistry, hydrothermal environments

## Abstract

The origin of life is one of the most fundamental questions of humanity. It has been and is still being addressed by a wide range of researchers from different fields, with different approaches and ideas as to how it came about. What is still incomplete is constrained information about the environment and the conditions reigning on the Hadean Earth, particularly on the inorganic ingredients available, and the stability and longevity of the various environments suggested as locations for the emergence of life, as well as on the kinetics and rates of the prebiotic steps leading to life. This contribution reviews our current understanding of the geological scene in which life originated on Earth, zooming in specifically on details regarding the environments and timescales available for prebiotic reactions, with the aim of providing experimenters with more specific constraints. Having set the scene, we evoke the still open questions about the origin of life: did life start organically or in mineralogical form? If organically, what was the origin of the organic constituents of life? What came first, metabolism or replication? What was the time-scale for the emergence of life? We conclude that the way forward for prebiotic chemistry is an approach merging geology and chemistry, i.e., far-from-equilibrium, wet-dry cycling (either subaerial exposure or dehydration through chelation to mineral surfaces) of organic reactions occurring repeatedly and iteratively at mineral surfaces under hydrothermal-like conditions.

## 1 Introduction

Studies related to the emergence of life, whether from the point of view of prebiotic chemistry, or from molecular biology, take place on the timescales of laboratory experiments, the lifetime of funded projects and funded students and postdocs. Each carefully crafted experiment aims at maximising the results obtainable on these timescales, each experiment adds to our accumulated knowledge and to the advancement of the field. However, no experiment has yet been run end to end, *i*.*e*. from the initial ingredients (organic molecules, liquid water, energy and essential elements, such as H, N, O, P, S, and transition metals) to the emergence of a protocell. Apart from the necessity of using stochastic chemistry, an acknowledged concept (Dass et al., 2016) but one that is difficult to put into practice, realistic timescales may not be conducive to “reaching the goal”.

This contribution does not aim to produce a simple formula to account for the emergence of life; rather, it seeks to describe the Hadean environment, when life emerged, in as realistic a way as possible to help guide future experiments. In addition to providing information on the prevailing Hadean rock lithologies, their mineralogy and chemical composition, we seek in particular to emphasise aspects of the environment, such as stability and longevity of particular conditions, which will constrain reaction rates for prebiotic chemistry. If a certain environment is envisaged as a possible prime locale for the emergence of life, how long was it stable, how long did it last? (Begging the question, how long did it take to go from simple molecular ingredients to the emergence of the first cell?). These concepts go over and beyond the very localised conditions in which stability would be death to prebiotic chemistry and where gradients and instability need to be the norm.

We take as outer boundary conditions for this study the consolidation of the Earth (4.54, Dalrymple, 2001, or ∼4.53 Ga according to Kleine et al., 2009; Connelly et al., 2012) and the oldest traces of life (an eternally controversial concept but, in our view, which will be explained below, certainly by 3.75 Ga and very probably before, e.g., 3.95 Ga). Note that the oldest preserved traces of life do not represent the first life forms. Occurring in Eo-Palaeoarchaean rocks (4.0–3.33 Ga) that formed after the likely emergence of life in the Hadean (4.53–4.0 Ga), these traces reflect an evolutionary stage that had already comprised chemotrophy and phototrophy by at least 3.45 Ga and possibly before (Westall et al., 2011a; Noffke et al., 2013; Hickman-Lewis et al., 2018). Within the bounds of these two critical dates is the establishment of habitable conditions on the Hadean Earth, by which we mean the appearance of water on the surface at temperatures conducive to prebiotic chemistry (e.g., Sleep et al., 2014; Zahnle et al., 2015).

Obviously establishing the dates of these critical time limits relies heavily on modelling and comparative planetology, as well as inherited geochemical signatures of former Hadean crust, inherited Hadean zircon crystals, and the few portions of Hadean (4.56–4.0 Ga), Eoarchaean (4.0–3.5), and Palaeoarchaean (3.5–3.2 Ga) crust that have been preserved. As a consequence, dating the emergence of habitable conditions is like a movable feast, it depends on the estimations of the models. These general considerations are perhaps less important than the relative stability and longevity of the location(s) where life may have emerged and that would necessarily have existed on much shorter time scales.

Our approach is from the global to the local scale in terms of the emergence of life. It is complementary to the approach taken by Saha et al. (2022) that regards the prebiotic processes leading to the emergence of life on the microenvironmental scale. In their recent review, Saha et al. note that, critical at these scales are the physicochemical properties of the substrates and different prebiotic reaction microenvironments on early Earth. These microenvironments comprise various kinds of liquid and mineral or aqueous environments. Examples of liquid microenvironments include aqueous ones (bulk aqueous solution, sea spray, gels, ice); non-aqueous liquids (solvents); e.g., organic acids; deep eutectic solvents, e.g., urea, glycerol, and acetamicide; high presuure super-critical fluids, e.g., CO2, H20; tar; the interiors of structures, such as lipid bilayers; condensed droplet microenvironments. Examples of the solid microenvironments include mineral surfaces, e.g., clay minerals, sulphides, phosphorous-bearing minerals, as well as the early mantle conditions, primarily its oxidation state, temperature and pressure.

## 2 Planetary formation and the condensation of water

Planetary formation is generally considered to have occurred about 4.54 Ga (Dalrymple, 2001) or ∼4.53 Ga according to Kleine et al. (2009) and Connelly et al. (2012), with our planet accumulating from pebble-sized materials rather than planetesimals and protoplanets, according to the latest models (Morbidelli et al., 2012; Johansen et al., 2021; Raymond, 2021). Nevertheless, continued accretion occurred and collision with another planet the size of Mars (Halliday et al., 1996) that occurred approximately 4.51 Ga (Barboni et al., 2017) and led to the formation of the Moon. Latest models suggest that this major collision was a double collision that took place at a relatively slow pace (otherwise Theia would have been drawn towards Venus) and at an angle of ∼45° (Emsenhuber et al., 2017; Asphaug et al., 2021). A slightly younger date for the collision, between 4.426–4.417 Ga, has been proposed by Connelly and Bizzaro (2016).

The consolidation of the Earth’s initial magma ocean, core formation and crustal differentiation is calculated by using various isotopic proxies (reviewed by Brasser et al., 2021) shown in Table 1. Core formation occurred between 4.45–4.53 Ga (see references in Table 1). Fractionation processes in the mantle/crust were perturbed by the Moon-forming impact, and the subsequent cooling of the planet would have been retarded by the huge, opaque CO_2_/H_2_0 degassed atmosphere that would also have contained additional volatiles from the molten crust/magma (Zahlnle et al., 2015). Zahnle et al. (2015) estimate that the post-impact atmosphere could have contained between 100–1,000 bars of H_2_O and CO_2_ that was augmented by smaller amounts of CO, H_2_, N_2_, various sulphur-containing gases, and other geochemical volatiles evaporated from the magma (Schaefer and Fegley, 2010; Fegley and Schaefer, 2013). In addition to the effects of a thick atmosphere, tidal heating caused by the closer Moon would have slowed down cooling of the mantle (Zahnle et al., 2015). Once internal heating decreased below the critical runaway greenhouse threshold, Sleep et al. (2014) estimate that the ocean could have condensed onto Earth’s surface under a CO_2_ atmosphere of about 100 bar. Initial temperatures of ∼200°C could have been too warm for prebiotic chemistry, however. If the upper temperature limit of life is taken as a benchmark, Sleep et al. (2014) calculate that surface temperatures reached about 122°C when the CO_2_ pressure decreased to approximately 25 bar. Critical to the establishment of habitable conditions (i.e. temperatures at which critical prebiotic chemistry could occur) at the surface of the Earth was therefore the removal of CO_2_ from the atmosphere, which occurred mainly by tectonic processes, modelled to have taken place on a time scale of about 100 My or more after the Moon-forming impact (Sleep, 2016).

**Figure 1:**
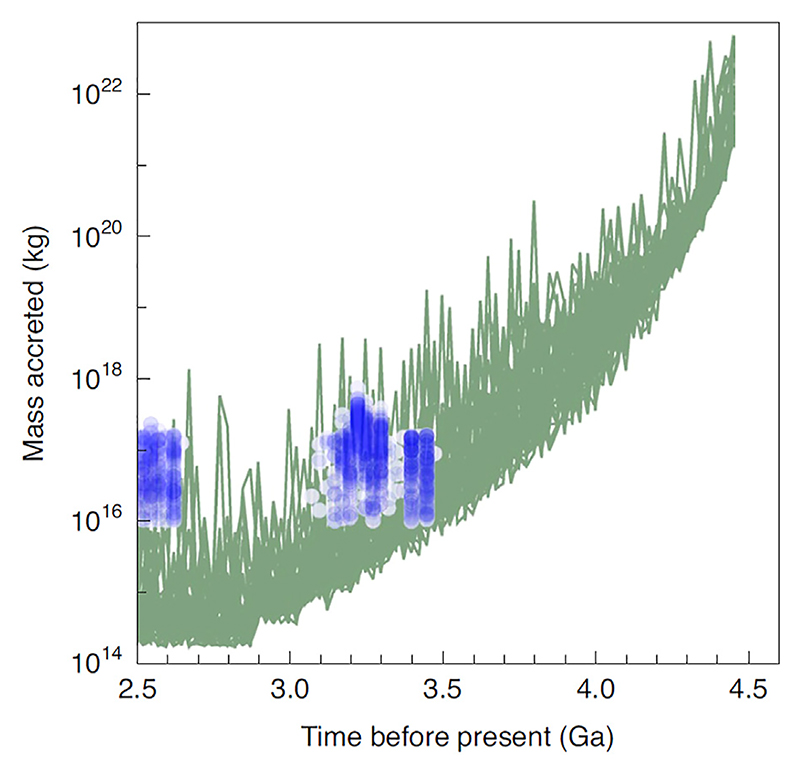
Earth’s collisional history based on modelling with the dates of Archaean impact spherule layers added (blue) (after Marchi et al., 2021).

Another factor of importance to take into account were the continued impacts, in particular those over 500 m diameter that could potentially have been planet-sterilising, while impactors over 300 m diameter would have increased surface water temperatures over about 100°C (Marchi et al., 2021). Their models suggest that the latest planet-sterilising impact occurred about 4.27 Ga. Figure 1 illustrates the modelled impact curve with known Palaeo-Neoarchaean impact spherule layers overlain (after Marchi et al., 2021).

### 2.1 Evidence for water and habitable conditions

There are a number of proxies for calculating whether or not there was liquid water at the surface of the Earth: 1) fractionated felsic crust, i.e. mafic crust that has been altered in the presence of water and fractionated to produce more silica-rich (felsic) crust; 2) zircon crystals of Hadean age that have?^18^O signature indicative of liquid water (i.e. formed in felsic melts); 3) sediments formed under aqueous conditions or specific mafic lava structures, such as pillow lavas, indicative of extrusion under water.

We will deal with felsic crust formation later because, owing to the absence of directly-preserved early crustal materials from the Hadean, we rely on inherited information from long-lived components, e.g., zircon crystals, that may have been formed either through fractionation of mafic mantle or during the production of aqueously-mediated felsic crust. These robust crystals generally have complicated histories often including overgrowths formed under different geological ages over the original crystal. Studies of the oxygen isotopic signatures preserved in Hadean-age zircon crystals (4.4–4.3 Ga, Wilde et al., 2001; Mojzsis et al., 2001; Valley et al., 2014) and combined oxygen and silicon isotope measurements (Trail et al., 2018) suggest that they formed in the presence of hydrothermal processing of the crust, implying the presence of water recycled into the crust from the surface of the Earth by 4.4 Ga. A number of more recent studies have questioned the age dating of the older zircons because of the fact that the crystals are long lived and exhibit multiple mantles of growth (Whitehouse et al., 2017), as well as the resetting of their U/Pb ages by later metamorphism or impact events (Griffin et al., 2014). What is important here is that the oldest, *bona fide*, oxygen isotope signature indicative of aqueous interaction with a fractionating felsic melt is revealed in a zircon aged 4.15 Ga (Muehlenbachs and Clayton, 1976; Whitehouse et al., 2017). This then is the baseline for liquid water at the surface, although, following the models described above, it is likely that liquid water at temperatures below ∼120°C existed previously.

Dating the oldest sediments known on Earth, i.e. detrital or chemical deposits formed in aqueous conditions, is highly controversial, as different dating techniques provide different ages. For example, the Nuvvuagittuq terrane in northern Quebec is purported to have a formation age of between 3.7 Ga according to U-Pb methods (Cates and Mojzsis, 2007) and 4.321 Ga according to the Sm-Ne method (O’Neill et al., 2012). This could be crucial because, if the terrane is Hadean in age, it holds the oldest known sediments. If not, it falls in the age range of the sediments from the 3.7–3.8 Ga Isua terrane in West Greenland (Appel et al., 1998), also containing metamorphosed sediments. In both cases, the sediments in question comprise banded iron formations, i.e. chemically (and possibly biologically) precipitated alternations of Fe-rich and Si-rich layers, as well as possible subaerial conglomerates. Volcanic sediments deposited under water are common in the younger, better preserved crustal remnants of the ∼3.5 Ga Barberton and Pilbara Greenstone Belts. Note that the Isua terrane contains the oldest pillow lava structures, i.e. evidence of direct lava extrusion under water. The rims of the pillow lavas are enriched in Fe_2_O_3_, MgO, MnO, K_2_O, Rb, Ba, Ga, Y, and transition metals compared to the cores (higher concentrations of SiO_2_, Na_2_O, P_2_O_5_, Sr, Pb, U, Nb, and the light rare earth elements (REEs) than the rims, Polat et al., 2003). Given the recent successful experiments to convert ribonucleoside triphosphates to polyribonucleic acid when incubated with the glassy rims of Hadean-analogue basalts (Jerome et al., 2022), these transition metal-enriched, vitreous rock surfaces are certainly very interesting for prebiotic chemistry.

### 2.2 Oldest potential and definitive evidence for life

The oldest morphological signs of life are purported to be hosted at the 3.7–4.3 Ga Nuvvuagittuq terrane. Dodd et al. (2017) and Papineau et al. (2022) report hematite filaments and tubes in the jasper-carbonate banded iron formation. The hematite filaments are centimetre-size, pectinate-branching, parallel-aligned, undulated, and contain Fe^2+^-oxides. Morphologically, they resemble modern Fe oxidising filamentous microorganisms. Papineau et al. (2022) invoke metabolic pathways including Fe-oxidation and S-disproportionation, as well as anoxygenic photosynthesis. However, given the great age and metamorphic condition of the purported traces of life it may be difficult to definitively conclude their biogenicity, and as pointed out by Papineau et al. themselves, the structures could be simply abiotic in origin. Indeed, these structures have been alternatively described as chemical precipitates because of their hydrothermal affinities (McMahon, 2019). Other studies have also demonstarted their non-biogenicity (Greer et al., 2020; Lan et al., 2022), describing the structures as metamorphic phenomena. Nevertheless, the fact that the sediment was clearly a chemical deposit indicates precipitation in water.

On the other hand, the oldest potential chemical signs of life during the Hadean comes from analysis of the carbon isotope signature of graphite in a 4.1 Ga zircon crystal of—24‰^13^C, consistent with biological fractionation (Bell et al., 2015). Similarly, the sediments in the 3.8–3.7 Ga Isua terrane hold potential evidence of life, also in the form of carbon isotope signatures (Rosing, 1999) as well as, more importantly, remnant organic molecules with compositions and structures suggestive of a biogenic origin (Hassenkam et al., 2017). The latter *in situ* FTIR investigation of metastable carbonaceous inclusions in a garnet crystal within the metamorphosed sediments documents structural binding of nitrogen, oxygen and possibly phosphorous to organic molecules, strong signatures that the material derived from living organisms. On the other hand, purported stromatolites from the same Isua Greenstone Belt (Nutman et al., 2016) are clearly metamorphic artefacts (Allwood et al., 2018; Zawaski et al., 2020).

Definitive evidence of widespread and varied life forms is well preserved in the ∼3.5 Ga Pilbara and Barberton Greenstone Belts. Both chemotrophic (Westall et al., 2006a; Westall et al., 2011a) and photrophic life forms (Hofmann et al., 1999; Allwood et al., 2006; Noffke et al., 2013) have been interpreted from the fossil remains. In the case of the Pilbara and Barberton Greenstone Belts, their excellent preservation has conserved morphological remains of microorganisms, biofilms, and stromatolites, as well as various isotopic and organo-geochemical evidence. The widespread distribution and degree of evolution of early life by 3.5 Ga strongly suggests its appearance much earlier (for a review of evidence of life and its implications and evolution in Southern Africa, see Hickman-Lewis and Westall, 2021).

Thus, by 4.15 Ga there is evidence of water at the surface of the Earth, as documented by zircon crystals of that age formed in a mantle environment influenced by altered, supracrustal material of felsic origin (Cavoisie et al., 2005). Apparently, a mechanism for fractionating carbon that was similar to that used by life today was in operation by then or soon after (Bell et al., 2015). By Isua times (3.8–3.7 Ga) life was very likely present on Earth, and by about 3.5–3.45 Ga it was widespread.

## 3 Rocky ingredients: Protocontinents, volcanic rocks (including sediments), hydrothermal silica

From the above, we conclude that the surface of the Earth was habitable before 4.15 Ga but, because of the uncertainties regarding timing of critical events, such as the Moon-forming impact, cooling of the planet, and removal of a large fraction of the CO_2_ from the atmosphere, that can only be addressed through modelling, it is not possible to be more precise in dating the initiation of habitable conditions.

In this section, we document what we know or think we understand about the Hadean/Eo-Palaeoarchaean crust in terms of protocontinents, and the evidence for exposed landmasses and fluvial input into the shallow water basins surrounding the exposed volcanoes. We document the composition of the volcanic rocks on the early Earth and detail the different kinds of environments that existed (for which we have evidence) or may have existed (for which we have no preserved geological record, e.g., deep ocean basins). We also emphasise the importance on the processes conducive to the origin of life of hydrothermal activity in the Hadean-Palaeoarchaean period, and of seawaters saturated in dissolved silica that precipitated out at different rates depending upon the environment, consequently exerting significant influence on the rates of prebiotic reactions.

### 3.1 Protocontinents and volcanic rocks

An important question regarding some of the possible scenarios for the origins of life is the availability of exposed landmasses. Models suggest that there was up to 40% more water on the surface (Sim et al., 2016), which was eventually slowly removed through plate tectonic recycling. It had originally been thought that the abundance of Hadean zircons indicated widespread production of felsic crust (and exposed landmass) through modern-style plate tectonics. This is not the place for a detailed discussion on the timing and origin of plate tectonics (a recent review can be found in Westall et al., 2022), but we will provide a brief overview here.

**Figure 2:**
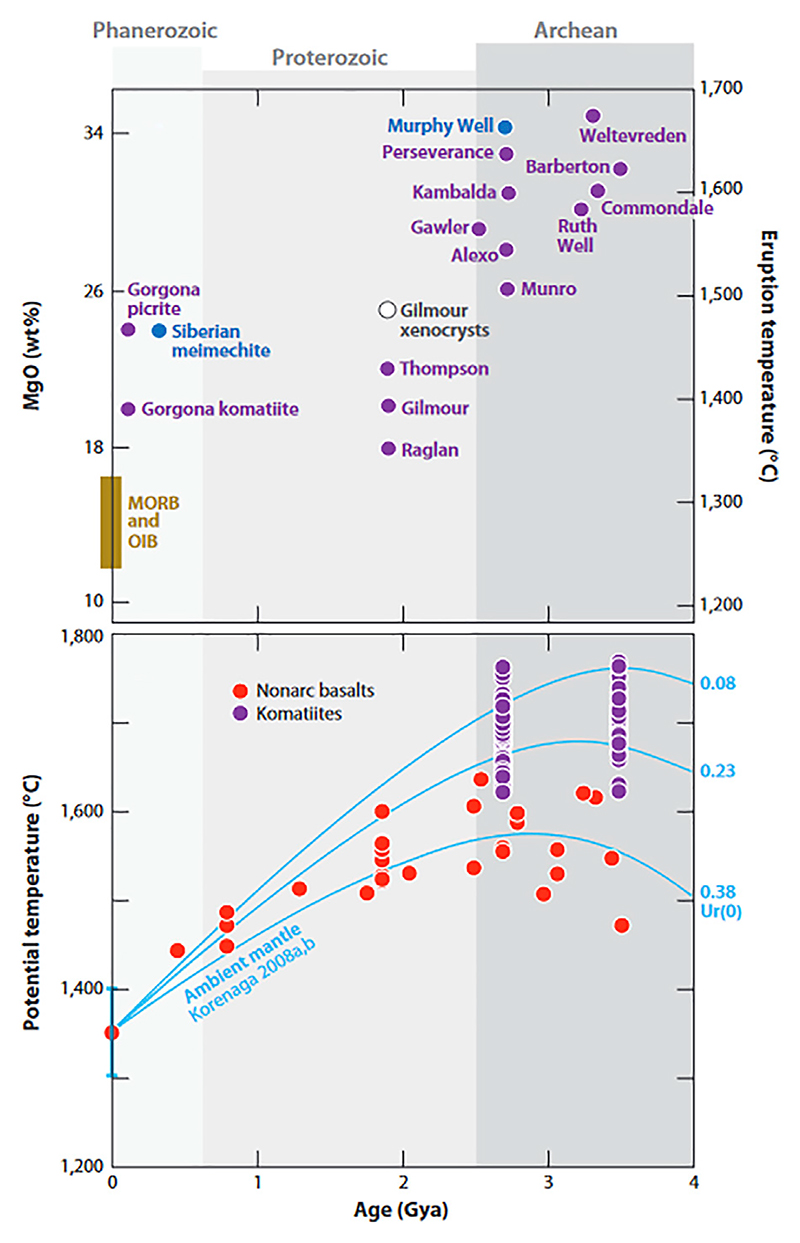
Eruption temperatures and mantle temperatures of komatiite lavas and melts through time highlighting the higher mantle temperatures during the Archaean and Hadean (after Arndt and Nisbet, 2012).

During the Hadean, when life emerged, the planet was dominated by higher mantle temperatures (Franck, 1998; Schubert et al., 2001; O’Neill et al., 2007; Perchuk et al., 2020), possibly up to six times higher than present day mantle temperatures. Indeed, ultramafic lavas, such as Mg and Fe-rich komatiites, common during the Archaean, are testimony to higher early mantle temperatures (Figure 2) (Arndt et al., 2008). The tectonic regime is uncertain but the Hadean Earth was likely dominated by a stagnant lid with likely squishy lid/plume-lid and plate tectonics (Sizova et al., 2015; Rozel et al., 2017; Lourenco et al., 2018), as well as some proto continents. Structural and geochemical evidence indicates that plate tectonics initiated between 4.0 and 3.0 Ga (Griffin et al., 2014; Lammer et al., 2018; Dehant et al., 2019), with some contending that modern style plate tectonics did not occur until only about 1 Ga (Korenaga, 2018; Hawkesworth and Cawood, 2020). The process would have been gradual and likely took place in different locations around the globe at different times.

Was there emerged continental crust during the Hadean? As mentioned above, the apparent relative abundance of Hadean age zircon crystals has been interpreted to suggest the formation of a significant amount of fractionated (felsic) Hadean crust (cf. Arndt and Nisbet, 2012). However, on the basis of cathodoluminescence investigations of zircon crystals to determine their age and history, Whitehouse et al. (2017) interprets many of the Hadean-age crystals as being younger, and the majority of them formed in the mantle rather than the crust. These authors noted four pulses of zircon formation centred around 4.37, 4.15, 4.1, and 4.02 Ga, but whether they indicate pulses in felsic crustal formation, enclaves of fractionated crust in a mainly mafic protocrust, or melts formed by impacts or other processes, is uncertain. Based on modelling and geochemical analysis (U–Pb ages, and ^177^Hf/^176^Hf ratios) of the ancient zircons, as well as Re–Os model ages on sulphides and alloys in mantle-derived rocks and crystals, Griffin et al. (2014) also conclude that the Hadean/Eoarchaean Earth was highly volcanic with rocks of predominantly mafic composition. Recycling of the crust would have been provoked by impacts, with fractionation occurring due to massive melting associated with huge impacts, similar to the Sudbury impact. Indeed, they also noted episodic clumping of zircon ages recording possible peaks in felsic crustal formation at 4.5 Ga (?); 4.2–4.3 Ga; 3.8–3.9 Ga;?3.3–3.4 Ga, possibly due to mantle overturns or major plume episodes, each followed by 150–300 Ma of quiescence.

Hadean protocontinents were not like modern continents. Modern continents are characterised by highly felsic cores, high elevations, and a thick, stabilising underplating “keel”. They are difficult to subduct. This was not the case during the Hadean. Since only highly metamorphosed remnants of Hadean protocrust survive, we will base our description on well-preserved crustal remnants dating from the Palaeoarchaean, the ∼3.5 Ga Barberton and Pilbara Greenstone Belts, that can be used, to a certain extent, as proxies of older crust. However, these ancient terranes record only remnants of the upper parts of protocontinents but no “deep sea crust” *per se*. They document abundant mafic and ultramafic effusions (massive and pillow basalts) and intrusions. Most of the volcanic lithologies are tholeiitic, calk-alkaline or komatiite-type basalts. The latter particularly are characterised by higher Mg contents than present day volcanics (by several orders of magnitude) (Table 2), and were formed from hotter melts (Arndt et al., 2008). The ultra/mafic volcanics may also be accompanied by more fractionated lavas or intrusions, including andesites, dacites and rhyolites. Table 3 documents the characteristics of Archaean basalts arand Arndt et al., 2008).

The Eoarchaean-Palaeoarchean continental crust was characterised by thick layers of mainly ultramafic to mafic lavas and intrusions and sediments derived from these materials, interspersed with rarer, more fractionated, felsic lavas and intrusions. Formation of the early granites (tonalite-trondjemite-granitoids, TTGs, less rich in Si and K compared to modern granites) intruding into this crust appears to have been related to lower crust melting rather than to plate tectonic subduction (Smithies et al., 2007). Indeed, structural and geochemical evidence of granites formed through plate tectonic subduction is not found in the Pilbara until after 3.2 Ga (Smithies et al., 2007).

Vanderhaeghe et al. (2019) suggest that the hotter Archaean crust was at least 40 km thick. Despite this, there is no evidence for widespread erosion of landmasses and exposure of the granitic cores of the early protocontinents, these were subdued landscapes with much of the continental crust submerged (Arndt and Nisbet, 2012). This means that the delivery of detrital material eroded from continents was lower than it is today. Thus, the early sediments comprised volcaniclastic detritus, either very locally derived from locally eroded rocks (the particles are generally euhedral to subhedral in shape, indicating lack of long transport by water, i.e. rivers), or ashfall into water bodies. These sedimentary materials were deposited into mainly shallow water basins and, although there is evidence of rare subaerial/deltaic fans, such evidence is rarely preserved, and one of the few examples is the 3.45 Ga Hoogenoeg Formation in Barberton (Lowe, 1999). Possible subaerial sediments have also been identified on the 3.75 Ga Isua and 3.8 Ga Nuvviagittuq terranes (Fedo, 2000; Bolhar et al., 2005; Cates and Mojzis, 2007; O’Neill et al., 2011).

**Figure 3:**
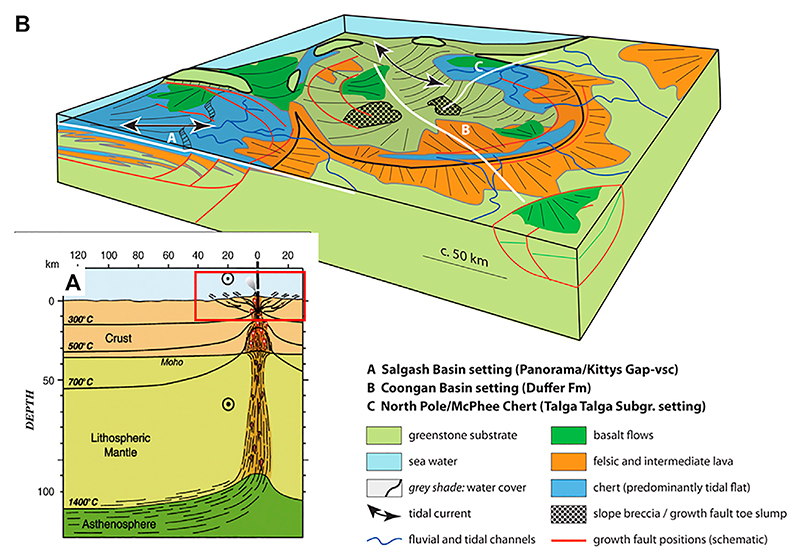
Schematic illustration of tectonic fracturing and basin formation on the early Earth. **(A)** A cross section of the early Earth’s crust and mantle highlighting crustal fracturing around the intersection between the mantle plume and the top of the crust (outlined by a red box) (after Vauchez et al., 2012). **(B)** Block diagram model of the upper crust, equivalent to the red box in **(A)**, showing the formation of collapse basins bordered by faults and fractures in the softer crust of the Hadean and Palaeoarchaean resulting in the formation of shallow water basins on top of the oceanic plateaux/protocontinents (after Nijman et al., 2017).

In a sedimentological-structural investigation of the basinal structures in the Pilbara Craton, Nijman and De Vries (2004) and Nijman et al. (2017) describe these features as collapse basins (Figure 3), similar to the coronae structures on Venus or early Mars (although not all researchers agree with this interpretation). These collapse basins are roughly circular in shape and of the order of 50 to several hundreds of kilometres in diameter. These basins were generally not very deep, since most sedimentary horizons were deposited in shallow settings (offshore to onshore), although some sedimentary sequences were deposited below wave depth (less than about 100 m). The basins formed on top of mantle plumes in the weaker (because hotter) Palaeoarchaean crust. Figure 3 illustrates collapse basins in the Pilbara Craton where the continental crust is mostly submerged, and only low relief volcanic edifices and surrounding areas emerged from the sea. In the example from the Pilbara Greenstone Belt shown in this figure, the emergent land masses are felsic volcanoes.

### 3.2 Hadean/Palaeoarchaean volcanic rock compositions

As representative examples of the mineralogical composition of Palaeoarchaean mafic lavas, tholeite basalts from the Kromberg Formation in the Barberton Greenstone Belt, South Africa, are comprised mainly of plagioclase (Na-Ca feldspar) and clinopyroxene (a Ca,Mg,Fe, Ti alumina silicate) with minor olivine and chromite (Vennemann and Smith, 1999) (Tables 2, 3). Titano-magnetites occur in the more evolved tholeites, while cumulates comprise only pyroxene and olivine. Komatiitic lavas (even more mafic than tholeites) are composed primarily of olivine and chromite (chrome spinels). They are characterised by anomalously high Mg (and Fe) contents owing to their very high temperatures of formation and eruption, up to >300°C hotter than basalts (Arndt et al., 2008). (Note that the original mineralogy of these Palaeaoarchaean basalts has been affected by seafloor alteration and subsequent metamorphism to actinolite-tremolite, chlorite, albite quartz, and rare, relict pyroxene.) Other Palaeoarchean basalt types include calk-alkaline basalts (enriched in magnesium and calcium oxides) comprise plagioclase, clinopyroxene and various metal oxides, such as magnetite.

In the Pilbara Greenstone Belt, there is much felsic volcanic material interlayered with the ultramafic and mafic lavas (Smithies et al., 2007). Here, more evolved lavas include andesite to dacitic rocks with sodic ratios of K_2_O/Na_2_O between .05 and .45 (van Kranendonk et al., 2007), as well as more enriched felsic rocks (low K_2_O < 1.0 wt%, high Fe, HREE and Y concentratinos positively correlated with SiO_2_ and La/Yb ratios) derived from fractionated tholeiitic lavas. Other felsic rocks have higher sodic ratios and have been derived from tonalite-trondgehemite-granodiorite (TTG) granites, while some highly potassic rhyolites may have been formed by remelting of pre-existing continental crust (van Kranendonk et al., 2007).

Examples of the compositions of andesitic (fine-grained lavas with phenocrysts of plagioclase and the ferromagnesian minerals, pyroxene and amphibole) to dacitic lavas from the Duffer Formation of the Pilbara Greenstone Belt (fractionated from basalts) are shown in Table 4. They include plagioclase porphyry (i.e. large plagioclase crystals (phenocrysts) in a fine-grained groundmass. Even more fractionated, porphyritic rhyolitic lavas occur in the Palaeoarchaean terranes, comprising more silica rich minerals, such as quartz and feldspars.

Typical of continental crust are granitic intrusions formed through fractionation of hydrated ultramafic to mafic crust, and sometimes remelting of previous granitic crust. In fact, the most common early crustal remnants are comprised predominantly of the early granitic cores of the protocontinents. However, as with the ultramafic lavas, the Hadean/Palaeoarchaean granites (TTGs) differed from their modern counterparts in their compositions, being less rich in potassium feldspathic minerals than modern granites (Moyen and Martin, 2013).

### 3.3 Volcanic sediments

While the early detrital sediments were mainly volcaniclastic in origin (including volcanic glass, protoliths, spherules, pseudomorphed feldspars and pyroxenes, as well as accessory minerals, such as chromites, zircon, rutile, quartz, carbonates, Fe oxides, and barite, see Lowe and Byerly, 2003), many of the sediments comprise varying amounts of carbonate minerals that formed diagenetically. All these lithologies have been significantly altered during early diagenesis to mostly Fe carbonate, sericite, chlorite, muscovite, and biotite followed by more or less complete replacement by silica, now forming the microcrystalline quartzitic rock, chert. With respect to the carbonates, given the diagenetic and metamorphic overprint, it is difficult to determine their original mineralogy, but Lowe and Knauth (1977) suggest local primary Fe-rich dolomite and ankerite for the Kromberg Formation in Barberton, now replaced by dolomite, siderite, ankerite and calcite. The Strelley Pool carbonates were Fe-dolomites (Shields, 2007).

Carbonates have also been described from the Isua Greenstone Belt in Greenland, but the calcites, Fe-dolomites and siderites are generally interpreted to be metasomatic deposits, although small amounts of carbonates associated with banded iron formations and metacherts may be original (van Zuilen et al., 2003).

### 3.4 Volcanic particle alteration

**Figure 4:**
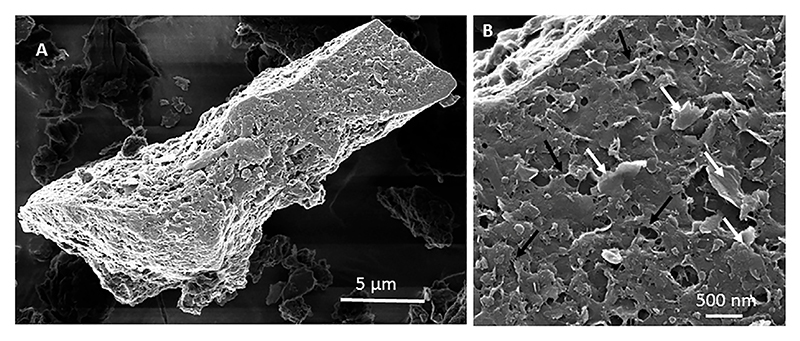
Alteration of the surface of a plagioclase fragment from crushed basalt by a Hadean seawater simulant at 73°C **(A)** showing rapid coating of the whole surface by minute clay particles **(B)**, white arrows. Note also that the mineral particle is coated with a “conditioning layer” of organic matter automatically precipitated from the ambient water (black arrows).

Mineral surfaces, such as clays, basaltic glasses, pyrite, or other reactive materials, are an important substrate for prebiotic reactions, as demonstrated by the recent conversion of ribonucleoside triphosphates to polyribonucleic acid when incubated with basaltic glass (Jerome et al., 2022). In the hot, generally acidic waters of the Hadean oceans, inorganic surfaces of volcanic materials or meteorites, would have been rapidly altered. For example, in the preserved Palaeoarchaean rocks, most of the volcanic detrital particles were altered to phyllosilicate (probably some kind of smectite, now mostly sericite, chlorite or muscovite after metamorphism) and to anatase before being silicified (Lowe, 1999; Foucher et al., 2010; Westall et al., 2015b). Moreover, an experiment to alter basalt and obsidian surfaces in artificial Hadean seawater at 73°C showed that the surfaces were rapidly coated with a mixture of clay particles and organic matter, the former apparently forming in association with the organic film (Figure 4) (unpublished data, Westall). In this experiment, the mineral surfaces were coated by a “conditioning film” of organic molecules as soon as the material was exposed to seawater. This is a known, automatic chemical reaction caused by hydrostatic interaction between the hydrophobic organic macromolecules and the hydrophilic surface of materials immersed into water, and it takes place within minutes (Costeron et al., 1995; Westall et al., 2000). All natural water contains dissolved organic matter (DOM), all biologically derived, in the reduced form. Indeed, Catala et al. (2021) note that DOM in marine waters has one of the most diverse molecular compositions known, consisting of millions of individual, low mass compounds, including compounds that are alicyclic, organic acids with amphiphilic properties (Hertkorn et al., 2006; Dittmar et al., 2008; Zark et al., 2017). While today much of the DOM is rapid recycled by life, some molecules have life times of >1,000 years. The latter situation would have been the case before the advent of cellular life in the prebiotic Hadean oceans. Today, it is estimated that there is more than 1 Eg (exagram, 1,018) of DOM on the planet (662 ± 32 Pg (petagram, 1,015) carbon; Hansell et al., 2009). DOM in the Hadean oceans would have been sourced from the mantle, hydrothermal crustal processes, and particularly from extraterrestrial meteorites and micrometeorites (Maurette, 2006). DOM from hydrothermal fluids would have been more common in the Hadean than today, given the higher crustal temperatures and more vigorous hydrothermal recycling. DOM in such fluids has been thermally altered (Rossel et al., 2017) and comprises more aromatic molecules and with less carboxyl-rich alicyclic species.

The close association of clay minerals with organic matter for prebiotic reactions has long been investigated (Cairns-Smith, 1966; Hartman, 1975; Ferris et al., 1979; Ferris et al., 1996). Interactions between the inorganic and organic phases are facilitated by adsorption, intercalation and cation exchange. Furthermore, negatively charged organic ions bind to clay minerals by positive edge charges or by the exchange of structural OH groups (Lagaly, 1984). Kloprogge and Hartman (2022) review present understanding with respect to the role of clay minerals, in particular mixed layer, Fe-clays, such as smectites, in the origin and development of metabolism, noting 1) the formation of amino acids on the surface of clay minerals on carbonaceous chondrites from simpler molecules, e.g., CO_2_, NH_3_, and HCN; and 2) the catalytic role of small organic molecules, such as dicarboxylic acids and amino acids found on carbonaceous chondrites, in the formation of Fe-clays themselves. Importantly, the evolution of metabolism can be replicated and catalysed by clays that can synthesise monomers, such as amino acids and nucleotides, that will subsequently polymerise, an important reaction for RNA-peptide worlds (op.cit.).

### 3.5 Hydrothermal activity and silica

One important component of these early sediments is chemically-precipitated silica (Dass et al., 2018; Westall et al., 2018; Ledevin et al., 2019). Hadean-Palaeoarchaean ocean waters were supersaturated in silica owing to global, widespread hydrothermal silica input (Hofmann and Harris, 2008), surficial weathering, and devitrification of the volcanic protoliths in water. Since chemical silica was deposited in rhythmic layers interspersed with other sediments, Ledevin et al. (2019) suggest that this was controlled by seasons, with silica precipitation occurring during winter months. The fact is that we have no idea what the yearly climate was like during the Palaeoarchaean era. Nevertheless, the rhythmic alternation of chemical silica layers with other sediment does indicate some kind of cyclical control, similar perhaps to that functioning for the banded iron formations.

One of the consequences of the early silica-saturated seawater was the pervasive silicification of all lithologies in contact with water, both sediments and volcanic rock surfaces. Silicification was extremely rapid in the vicinity of hydothermal activity in these facies (Westall et al., 2015b; Westall et al., 2018), and slower away from the direct influence of hydrothermal vents, where there is evidence of more advanced diagenesis. The degree of silicification and its rapidity has important consequences for prebiotic chemistry, as discussed below.

**Figure 5:**
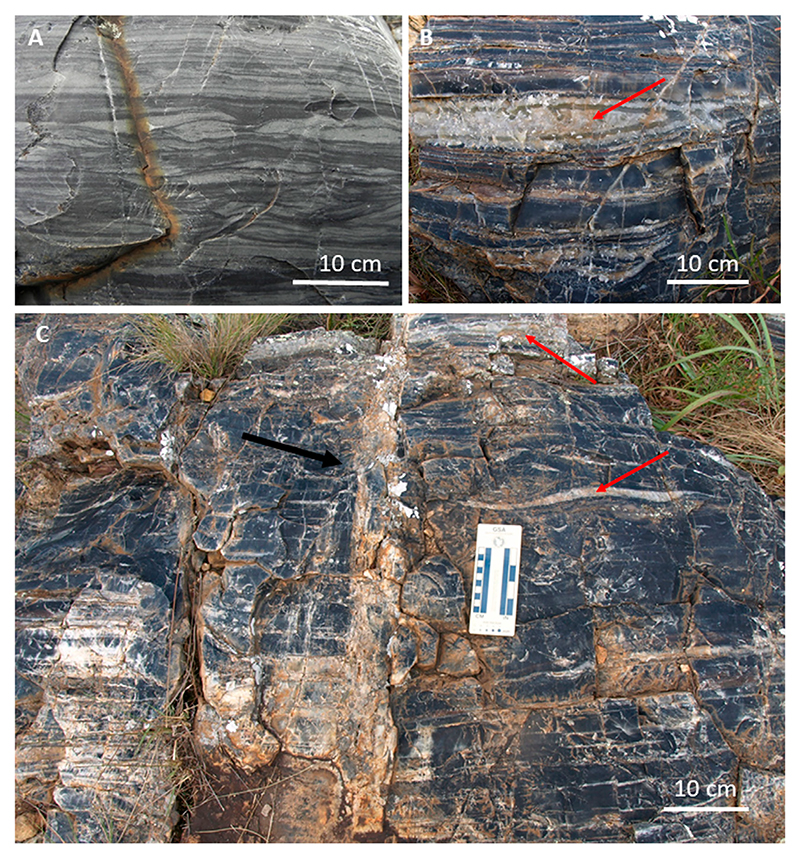
The effects of silicification on volcanic sediments, examples from the 3.33 Ga Josefsdal Chert, Barberton Greenstone Belt, South Africa. **(A)** Layers of volcanic ashfall deposited in a littoral setting and away from direct hydrothermal influence with a total silica concentration about 96% (@J.Bréhéret). **(B)** Similar sediments deposited close to hydrothermal activity with a total silica concentration >99.9%. Note the bedding parallel hydrothermal infiltration and possible vadose crack indicating subaerial exposure (red arrow). **(C)** Hydrothermal vein traversing the horizontally laminated shallow water sediments (black arrow) and lateral infiltrations of hydrothermal fluids into already slightly consolidated sedimentary layers (red arrows).

White smoker-type hydrothermal vents, with their associated carbonate deposits, are not recorded in the crustal record of Eo-Palaeaoarchaean cratons. They were either rare, did not exist, or were not preserved. They may have existed in the Hadean deep oceans. What do exist in the geological record are silica-rich vents that expulsed silica-rich fluids and, in the process, thoroughly silicified the surrounding lithologies (Hofmann and Harris, 2008; Westall et al., 2015a), forming pods of sediment around the conduits that are now >99.9% SiO_2_ (Figure 5). Silicification implies permeation of all matter, particulate, solid rock or biogenic, by silica and replacement of the pre-existing feature by silica. Replacement may be more or less complete, leading to ghost structures; or partial, depending upon the degree of saturation of the seawater by silica. Other factors, such as temperature and pH, will have an effect on the rate of silicification.

One way to estimate the rate of silicification is the degree of diagenesis of the non-organic detrital and biogenic facies. Close to hydrothermal effluents, biogenic remains were so rapidly silicified that there was no time for diagenetic alteration that would lead to the precipitation of framboidal pyrite (sometimes associated with biogenic activity, such as degradation of pre-existing organic matter, e.g., dead cellular materials, by heterotrophic microbes, such as Sulphur reducing bacteria (SRBs), e.g., Duverger et al., 2020; Duverger et al., 2021). At further distance from hydrothermal activity, framboidal pyrite associated with degraded organic matter was common (F. Westall, personal observations). In an experiment to document the formation of pyrite in a pure microbial culture of sulphate reducing bacteria, Duverger et al. (2020) noted that spherules of pyrite a few hundreds of nm in size precipitated after 1 month of incubation. Indeed, pyrite framboids have not been described in association with well-preserved, cellular fossils from the Palaeaoarchaean sediments, confirming their rapid silicification (Westall et al., 2001; Westall et al., 2006a; Westall et al., 2006b; Westall et al., 2011a; Westall et al., 2011b). Superb cellular preservation by silicification is another means of estimating the time scales of silicification. The Palaeoarchaean cells mentioned above document preservation of intact cells undergoing cell division, as well as cells and colonies in varying stages of degradation.

Experimental silicification of the kinds of thermophilic Archaea that could have existed on the early Earth, at temperatures of 60°C documents initiation of fossilisation already within 24 h (Orange et al., 2009). Note that the Palaeoarchaean shallow seas were very warm, ∼50°C–76°C, water temperatures being estimated from oxygen and silicon isotopes, e.g., van den Boorn et al. (2007), Tartèse et al. (2017), and Lowe et al. (2014) In these cases, the organic substrates functioned as substrates for the chelation of silica, either directl.y onto the organic matter, or through cation bridging, e.g., with Fe. This was rapidly followed by direct chemical precipitation of silica in an acidic environment that formed a matrix cement around the organic substrate. Initially the silica would have presented a gel-like consistency that permitted permeation of fluids (Iler, 1979) and prebiotic reactions. As it polymerised and dehydrated, this permeable capacity would be lost. The rate of polymerisation of silica into silica gel depends on the saturation of silica, pH and temperature, as well as the presence of dissolved salts (Iler, 1979). We can conclude that, close to hydrothermal activity in the salty (Knauth, 2005, estimates early salinity to be 1.5–2.0 times present level because of the relative paucity of continents and enclosed water bodies in which halite could precipitate, consistent with estimates for salinity also on early Mars in Fairén et al., 2009), warm to hot environment of the early Earth, initial precipitation of silica was on the order of hours to days, while further polymerisation and dehydration leading to the cessation of prebiotic chemical reactions taking place within the gel, would have occurred on longer time scales. Further away from direct hydrothermal influence, the rates of precipitation, polymerisation and dehydration would have been slower (time scales of one to a few months, cf. Duverger et al. (2020)), as documented by the more prolonged diagenetic alteration of the organic matter leading to framboidal pyrite precipitation.

The silica formed initially by polymerisation of monomeric silica in solution, nucleating to form ever larger particles depending upon the availability of the silica. The supersaturated Palaeoarchaean seawater thus precipitated silica, both within the sediments as well as on top of them forming layers of chemically precipitated silica gel (cf. Ledevin et al., 2019). Evidence of this chemically precipitated gel formation is seen in where colonies of microbes grew in three-dimensional spicular colonies within a mixture of fine volcanic dust and silica gel (Westall et al., 2015a; Hickman-Lewis et al., 2020a). Again, in these cases, the rarity of pyrite associated with the degraded, silicified organic matter is testimony to the rapidity of silicification.

The fact that silica gel was pervasive in the Palaeoarchaean oceans has important consequences for prebiotic reactions. Silica gel is a porous medium. Incorporating reactive volcanic detritus (including primary minerals, such as (rare) olivine, pyroxene, plagioclase feldspar, oxides e.g., chromite, and pyrite, and secondary minerals (alteration phases), such as clays, carbonates, anatase), the silica gel could act as myriads of microscopic reactors, especially when influenced by hydrothermal effluent (Dass et al., 2018; Westall et al., 2018). Here, gradients in pH, ionic concentrations and temperature from the vents through the immediately surrounding sediments would have been important factors for prebiotic reactions taking place in these sediments. Indeed, Pollack (2001), Trevors and Pollack (2005) and Dass et al. (2018) note that gels represent an excellent mechanism to maintain concentration gradients, alter the structure of water, and influence ion-macromolecule interactions. Gradients of protons and cations are conferred because the ability of gels to exclude solutes maintains the overall system out of equilibrium by creating an imbalance of solutes inside and outside the gel.

The rate of silica gel formation and its subsequent dehydration and lithification would have been an additional control on rates of prebiotic reactions. For example, using an artificially prepared solution of silica (sodium silicate) in artificial Hadean seawater, Dass et al. (2018); Dass et al. (2018) showed that the gel can form within hours upon addition of glacial acetic acid (pH 2.5). Of course, in the warmer Hadean waters, where pH would have been variable at the surfaces of the volcanic rocks and sediments (initially a relatively high pH 8, then decreasing after a couple of days to pH 6, Westall et al., 2018), the rate of silica gel formation would also have been influenced by the saturation of silica in the ocean waters, which, as we noted above, appears to have been controlled also by volcanism and direct hydrothermal input. Rapid silica gel formation would have occurred during the Hadean-Palaeoarchaean over time spans of hours to possibly days. With pore sizes of the order of 100 s nm to a couple of micrometres, these pervasive gels were a critical physical and chemical component of the early rock/sediment interfaces.

While the silica gel was in a porous state that allowed the transport of fluids, small molecules and dissolved components, prebiotic reactions could take place within the precipitate. However, dehydration and solidification of the gel that would put an end to these kind of reactions would depend on factors, such as temperature and pH. For example, Gallo and Klein (1988) note that silica gel dehydration is constrained by the diffusion of hydrogen-bonded species at low temperatures (<600°C), as would have been the situation in the Hadean oceans. Gels formed under basic conditions are structurally different to those formed under acidic ones (Orcel et al., 1986), the former being characterised by a macroporous structure with surface silanols and the latter by a microporous one with internal silanols. Acid-formed gels have a smaller scale silica network, larger surface area, smaller pores and larger pore interconnection. However, dehydration of silica gels formed under basic conditions is more rapid than those formed under acidic conditions. Sleep et al. (2001) and Sleep (2010) consider that the Hadean oceans were probably as neutral as they are today because of buffering of the seawater chemistry due to reactions with basalt. However (Ueda et al., 2021), suggest that fluids close to hydrothermal vents would have been probably more acidic at low temperatures owing to high water/rock ratios during the hydrothermal reactions (although pH would have been more alkaline at higher temperatures). Also, the predominantly CO2 atmosphere would have resulted in slightly acidic waters, as equally proposed for early Mars (Fairén et al., 2004). As noted above, while more alkaline alteration of the surfaces of Palaeoarchaean subaqueous basalts occurred at high temperatures (e.g., Robins et al., 2010), the pervasive silicification of the sedimentary horizons and the tops of the basaltic sequences is testimony to a lower temperature, silica-rich, acidic medium.

### 3.6 Fluvial input

**Figure 6:**
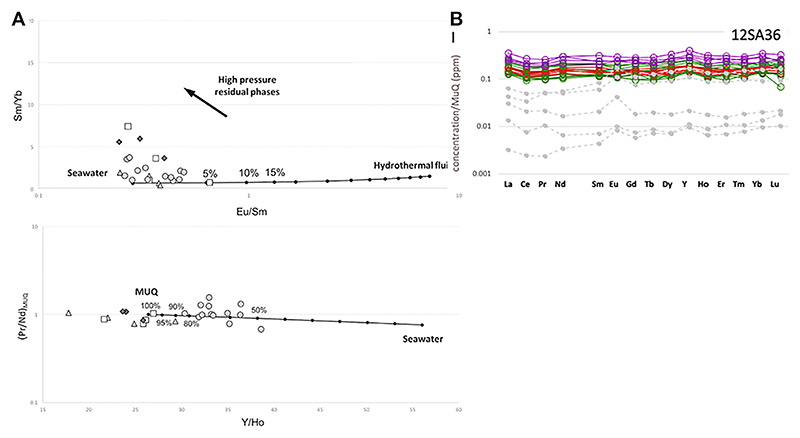
Geochemical evidence for seawater, hydrothermal and riverine signatures in the Palaeoarchaean shallow seas (the Josefsdal Chert, Barberton greenstone Belt). **(A)** The Sm/Yb and Eu/Sm ratios show a distinct hydrothermal influence, while the Pr/Nd and Y/Ho ratios document a distinct terrigenous influence **(B)** While bulk measurements of the Josefsdal cherts show the La, Eu and Y anomalies typical of hydrothermal fluids, *in situ* measurements document the flat REE distribution indicative of terrigenous, i.e., riverine input (After Hickman-Lewis et al., 2020b).

In the Barberton craton, the situation is similar (Nijman and de Vries, 2004). As noted above, most of the sedimentary detritus derived from exposed volcanic lavas and volcanic ashfall. Evidence for riverine input into the shallow basins comes from geochemical data. Hickman-Lewis et al. (2020b) made *in situ* LA ICPMS measurements of microbial mat layers (i.e. relatively short-lived, ephemeral phenomena) that document flat, light REE-enriched REE + Y patterns and chondritic Y/Ho ratios indicating major contributions from terrigenous, riverine fluids, i.e. continental weathering, on a short temporal scale. This signature is superimposed on a long-term, background signature of predominantly hydrothermal origin (Figure 6).

### 3.7 Solar irradiation

**Figure 7:**
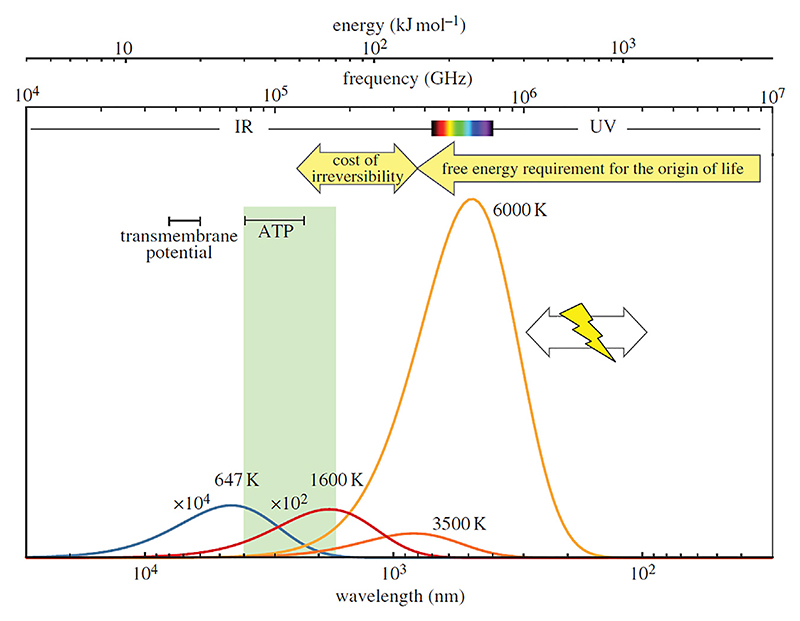
Free energy source requirements in living systems (after Pascal et al., 2013). The energy available at the critical point of water (at 647 K) is shown by the blue line; typical Hadean magma temperatures (∼1600 K) are in red; the surface temperatures of examples of M-stars (3500 K) or G-stars (e.g. Sun, 6000 K) in dark and light orange lines; and lightning (T ≥ 104 K). Pascal et al. (2013) note that a much higher potential [ca 150 kJ mol–1 (42,43)] than the free energy potential of common biochemicals (green rectangle 30–70 kJ mol–1, including ATP) was required to trigger the self-organization of life after taking into account the cost of irreversibility (yellow arrows), namely visible and UV light, as well as lightning.

Numerous different types of locations have been suggested for the emergence of life (see review in Westall et al., 2018). The most commonly considered are hydrothermal environments but locations including associated with impact craters (Osinski et al., 2020; Sasselov et al., 2020), pumice rafts (Brasier et al., 2011; Brasier et al., 2013), deep seated fault zones (Schreiber et al., 2012), and radioactive placer sands (Adam et al., 2018) have also been invoked. For many of these environments, solar irradiance at the surface of the Hadean Earth, both on exposed landmasses and in water bodies, could have played an important role in the emergence of life. Pascal et al. (2013) hypothesise on the possible importance that UV radiation may have had as a free energy requirement for the origin of life (Figure 7), although not all prebiotic chemists adhere to this theory. Modelling suggests that solar irradiation at the surface of the early earth was much higher than today.

Solar X-ray and UV radiation covers wavelengths from .1 to 320 nm. The luminosity of the Hadean/Palaeoarchaean Sun was about 74%–77% of its present value (Bahcall et al., 2001) but the solar X-ray and UV (EUV and FUV) luminosity reaching the top of the early Earth’s atmosphere was higher (Micela et al., 1985; Micela et al., 1988; Micela, 2002; Cnossen et al., 2007). Radiation at the surface of the early Earth would have been controlled by the composition of the atmosphere. Cnossen et al. (2007) modelled the spectral radiance of the young Sun on the basis of evolution of solar-like stars, taking into account factors, such as atmospheric composition, solar flares and activity cycles, as well as aerosol concentrations and cloud cover. They showed that shorter wavelength radiation (<200 nm) would have been attenuated by the atmosphere, while the flux of longer wavelengths (>200 nm) reaching the surface of the Earth would have been ∼10^–5^ Wm^−2^ nm^−1^ at 200 nm, to ∼.4 W m^-2^ nm^−1^ at 320 nm for small concentrations of CO_2_ in the atmosphere (.024 bar). Higher CO_2_ atmospheric concentrations (e.g., 1.20 bar) would have significantly attenuated the surface flux of radiation (∼10–16 W m^-2^ nm^−1^ at 200 nm, to .02 W m^-2^ nm^−1^ at 320 nm. Cnossen et al. (2007) calculated the irradiance at the top of the atmosphere and the surface irradiance (180–320 nm because the shorter wavelengths do not reach the surface) for the early Earth (Archaean in this modelled case, i.e. 4.0–2.45 Ga) at different atmospheric CO_2_ concentrations, compared with the DNA weighted spectra for different CO_2_ concentrations (Cnossen et al., 2007). also demonstrated that small amounts of other gases in the atmosphere, such as N_2_, CO_2_, CH_4_, H_2_O, O_3_, O_2_, and SO_2_ would have little effect on the attenuation of solar irradiation. They conclude that the amount of radiation reaching the early Earth’s surface would have been damaging for the DNA of early life. From the point of view of Pascal et al. (2013), this high radiation is essential for providing the necessary initializing energy for prebiotic reactions in order for the “multiplying entities (to be) associated with the dissipation of free energy” (Figure 7).

Solar radiation is, however, generally rapidly attenuated by water, reaching at maximum depths of a few metres, although note that blue-green light (UVA at 360 nm) can penetrate up to depths of 50–70 m in oceanic waters when the Sun is at its zenith (lee et al., 2013). Attenuation of radiation at all wavelengths will be increased depending upon water opacity. On the volcanically-active early Earth, water bodies would have carried much suspended and sedimenting particulate matter (volcanic debris, e.g., ashfall and detrital sediments) during eruptive phases, which would have significantly decreased solar radiation penetration depths. The fluvial contribution to the solid particulate load being deposited in shallow water basins adjacent to the exposed volcanoes would also have been higher during and after eruptions owing to the increased topography and erosion of the volcanic edifices. However, the intervening quiescent periods would have lower detrital input and hence suspended sediment load.

## 4 Characteristics of hypothesised locations for the emergence of life

Numerous different types of locations have been suggested for the emergence of life (see review in Westall et al., 2018). The most commonly considered are hydrothermal environments but locations including associated with impact craters (Osinski et al., 2020; Sasselov et al., 2020), pumice rafts (Brasier et al., 2011; Brasier et al., 2013), deep seated fault zones (Schreiber et al., 2012), and radioactive placer sands (Adam et al., 2018) have also been invoked but in this section we will concentrate on hydrothermal environments.

Of importance for any of the hydrothermal environments described below are gradients present, some of which, such as redox gradients, can fuel prebiotic reactions (Villafañe-Barajas and Colín-García, 2021). The various gradients include, temperature, solute concentrations, density, pH, as well as redox gradients. Reduced carbon compounds (e.g., HCOOH and CH_3_OH) have been produced *via* thermal gradients and reversible reactions between dissolved gases (i.e. CO_2_, CO and H_2_) (Seewald et al., 2006). Moreover, it has been suggested that temperature gradients and oxidation–reduction reactions can contribute to the synthesis of organic molecules from gases, such as CO_2_ and H_2_ (Shock, 1993; Shock and Schulte, 1998; McDermott et al., 2015). Note, however, that for some metals, such as Fe and Mn, redox reactions can be kinetically slow (McCollom, 2000). Nevertheless, a recent experiment has shown that dipeptides may be formed by interaction of amino acids with minerals, such as olivine and orthopyroxene (Takahagi et al., 2019).

### 4.1 Subaerial springs

Recently, subaerial springs have been proposed as suitable locations for the emergence of life (van Kranendonk et al., 2018; Damer and Deamer, 2020; van Kranendonk et al., 2021). Today, subaerial springs are common in volcanic areas associated plate margin and intraplate, long-lived (10 s My) plume activity. During the Hadean, plumes on the hotter early Earth may have existed for longer, up to several 100 s Ma, judging by the ages of the Palaeoarchaean greenstone terranes, while individual volcanic eruptions and associated hydrothermal activity would have been more short-lived and episodic. For example, hot spring activity at Yellowstone over the North American mantle plume has been ongoing since at least the end of the last ice age (∼15,000y) with the construction of large geyser cones taking place over thousands of years (USGS, 2022). A modern analogue of Hadean/Palaeoarchaean protocontinents with associated volcanic and hydrothermal activity is the Kerguelen plateau with its volcanic island archipelago. Characterised by mainly basaltic lavas with some felsic components intruded by plutonic rocks, the volcanic rocks date back to 39 Ma (Gagnevin et al., 2003), with individual lava complexes have a longevity of a couple of million years.

**Figure 8:**
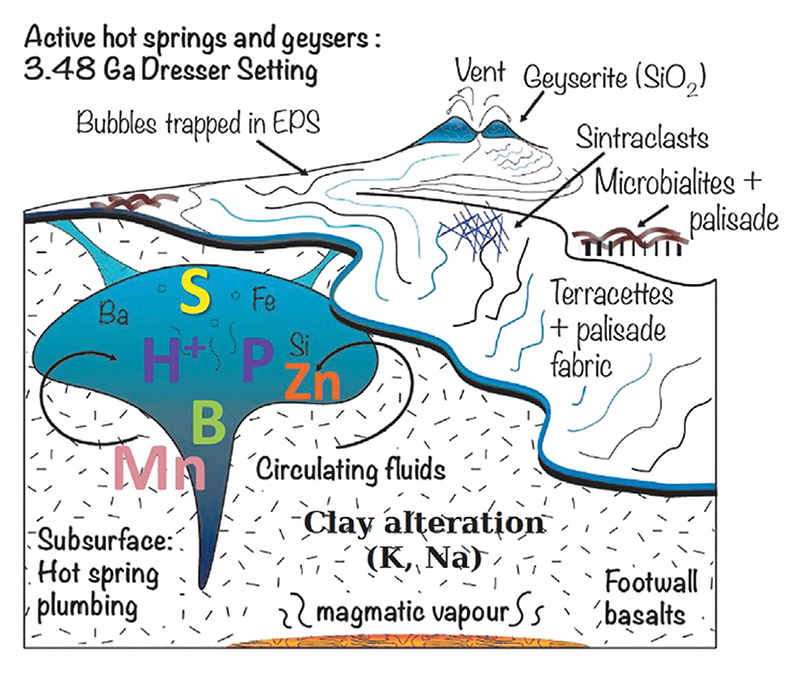
Schematic representation of the Dresser hot spring system and the elements it concentrated in hot spring pools and in the altered footwall (after van Kranendonk et al., 2021).

Palaeoarchaean suberial springs have been described from the Pilbara Greenstone Belt, while much of the hydrothermal activity in the 3.48 Ga Dresser Formation occurred under shallow water submarine conditions. In one case, some of the venting was associated with relatively restricted (<10 m) geyserite and siliceous sinter deposits in the North Pole Chert, typical of a subaerial hot spring field (Djokic et al., 2017; Van Kranendonk et al., 2018; Djokic et al., 2021) (Figure 8). Van Kranendonk et al., 2021 and references therein) describe a volcanic caldera setting characterised by voluminous, contemporaneous hydrothermal fluid circulation that formed a dense network of hydrothermal chert and baryte veins cutting through the underlying komatiitic pillow basalts and lowermost layers of the North Pole Chert. Van Kranendonk et al. (op.cit.) conclude that the sedimentary succession records “a transition upward from open marine, through a shallow, evaporative marine basin (caldera phase), to subaerial conditions with hot springs and fluvio-lacustrine deposition, followed by a return to deep marine conditions that resulted from caldera collapse with/without active extension (Djokic et al., 2017; Van Kranendonk et al., 2018; Djokic et al., 2021)”.

The hydrothermal veins associated with the North Pole Chert succession are large, up to 50 m wide and also deep, penetrating down to depths of several kilometres. The tops of the veins are associated with the hot spring deposits and mineralised remnants of the hot spring pools (Van Kranendonk et al., 2007; Djokic et al., 2017; Van Kranendonk et al., 2018; Djokic et al., 2021). Fluid temperatures in the veins are estimated to have been about 350°C at depth and about 120°C at the palaeosurface (Harris et al., 2009). Interestingly, tourmaline crystals (containing boron) are associated with some of the hydrothermal deposits, and boron is of interest in prebiotic processes (cf. Ricardo et al., 2004), as noted above.

Although the individual Dresser springs were of limited dimension (Kranendonk et al., 2021), it is known that subaerial springs may occur in swarms of many hundreds or even thousands of individual vents, each with different physico-chemical parameters in terms of temperature, fluid chemistry (pH, ionic concentration, element composition), gas content, style of upwelling (passive flow, to bubbling, to geysers), that can vary widely over lateral scales of only a few meters (Campbell et al., 2015). These kinds of environments provide a wide range of possibilities for concatenating prebiotic reactions over periods of up to a couple of million years, according to the Kerguelen analogy.In an analysis of the chemical energy available at one subaerial spring (Obsidian Pool, Yellowstone), Shock et al. (1995) determined that the energy yields for different redox reactions involving reduced hydrothermal fluids depend largely on the electron acceptors. In this modern case, the electron acceptors include, O_2_, nitrate, nitrite, elemental S, magnetite, hematite, goethite, sulfate, CO, and bicarbonate/CO_2_ in order of the highest to lowest energy yields. On the Hadean Earth, O_2_ would only have been available in very small quantities, formed by abiotic radiolysis of H_2_O. Likewise, nitrate and sulfate were probably not present, and nitrite limited.

### 4.2 Submarine environments

Scenarios for the emergence of life in the submarine realm are mainly related to hydrothermal activity (Baross and Hofman, 1985; Russell and Hall, 1997; Martin et al., 2008). The submarine realm on the Hadean/Palaeoarchaean Earth comprised a wide range of environments with differing physico-chemical characteristics, and hosted above differing types of igneous substrates. These include the shallow water, volcano flanking environments on top of the oceanic plateaux that were the protocontinents (i.e. at littoral to subwave base water depths), as well as environments in the deep ocean associated with plume-related hot spots and, if they existed in the Hadean when life emerged, early tectonic spreading ridges. Redox reactions, such as those described at the beginning of Chapter 4 involving dissolved gases (e.g., CO_2_, and H_2_) (Shock, 1993; Shock and Schulte, 1998; McDermott et al., 2015) or metal sulphides as catalysts and H2S as a reductant (He et al., 2019) to form reduced carbon compounds (e.g., formate and acetate in the latter case).

#### 4.2.1 Deep sea hydrothermal environments

**Figure 9:**
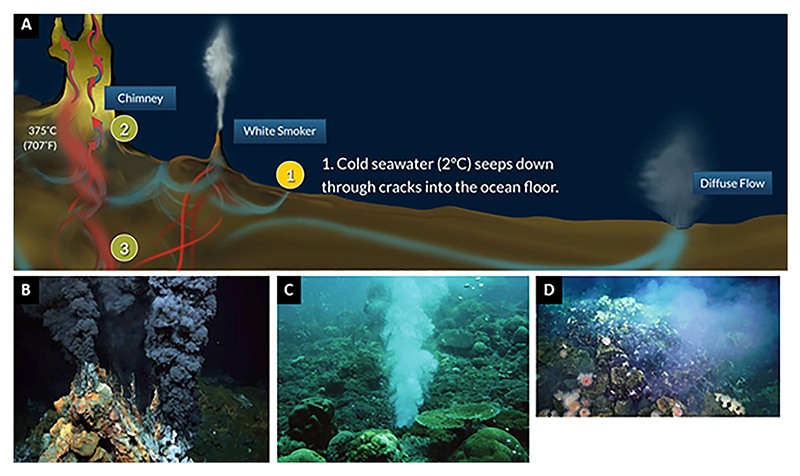
Deep sea hydrothermal vents today. (A) Sketch showing high temperature black smoker point sources (>350°C) on ridge axes, slightly lower temperature white smoker point sources (<300°C) off ridge axes, and more diffuse, low temperature (<100°C) sources further away from ridge axes. (sourced from the Woods Hole Oceanographic Institution). (B). Black smoker vent (after Rogers et al., 2015). (C) White smoker vent (after Karlen et al., 2010). (D) Diffuse venting (sourced from Seveseas.media.org).

There is a wide variety of hydrothermal systems forming in the deep sea environment, their physico-chemical characteristics controlled ultimately by the underlying igneous rock (and sediment) compositions, heat flow, and volcanic activity (Figure 9). They may be associated with actively spreading ridges and differ in style depending upon rate of ridge spreading, or they may occur at some distance from ridge axes, as well as above mid plate (or ridge/hot spot associations) mantle hot spots associated with submarine volcanism or seamounts. The hydrothermal systems may produce focussed vent edifices, as in the black smokers or some white smokers, or they may eject fluids in a more diffuse manner, especially for lower temperature systems that have a relatively strong component of intermixed seawater. Each style of venting, each hydrothermal field has different spatial dimensions and different longevity of activity, often with episodic activity. We have noted above that there is no preserved deep sea crust from the Hadean/Palaeoarchaean. However, hot spot activity and perhaps some form of early plate tectonics with spreading ridges, subduction and back arc basins may have been present (although there is a current consensus that plate tectonics did not start until after about 4 Ga, see Lammer et al., 2018; Dehant et al., 2019, or even later). Below we review the salient characteristics of the different types of deep-sea hydrothermal activity that could have been active during the Hadean.

Present day studies concerning the emergence of life in hydrothermal locations tend to concentrate on those representing processes occurring at or near high temperature (up to 400°C), acidic (pH 2–5), Fe-rich black smoker type vents that characterise spreading ridges (Corliss et al., 1981; Russell and Hall, 1997); and cooler, acidic Zn-rich white smoker vents (<300°C), also forming at or near ridge axes; or the even cooler (40°C–90°C), alkaline (pH 9–11), carbonate-rich vents (e.g., Russell, 2007; Martin et al., 2008) that form at some distance from the spreading ridges and emit hydrogen and methane. (Both the lower temperature white smoker and the even cooler alkaline vents are often referred to as “white smokers” in the literature). The latter environments are presently considered to be more likely for abiogenesis (Martin et al., 2008). These are environments characterised by well defined, focussed vents, as well as more diffuse venting in the case of the white smokers. However, hydrothermal activity in the deep oceans is not just confined to ridge axes and areas off the ridge axes, it also occurs wherever there is volcanic activity, e.g. hot spots above mid plate mantle plumes or island arc calderas, for example. Note that, in this context, there is a significant amount of fluid flow (of a variety of temperatures) in three dimensions for up to hundreds of kilometres away from the spreading centre. This is demonstrated by the fact that the majority of the advective heat loss associated with the ridges actually occurs on their flanks, as well as in older crust and at lower temperatures than on-axis systems (Stein and Stein, 1992; Stein and Stein, 1994). This activity results in significant off-ridge hydrothermal circulation resulting in circulation of solute enriched fluids between the crust and ocean (e.g., Elderfield and Schult, 1996; Wheat and Mottl, 2004; Fisher and Wheat, 2010) and geochemical processes within the crust (e.g., Boschi et al., 2006; Bach and Früh-Green, 2010).

Black smoker vents occur in fields of many individual exit points directly associated with volcanic activity and distributed on scales of meters to tens of kilometres. They may be associated with point sources, or along linear, ridge-related fissures (Figure 9). In any one field, some vents may be active while others may be extinct. Pulses of hydrothermal activity will follow pulses of volcanic activity. Haymon et al. (2008) conclude that the life span of black smoker-type hydrothermal activity related to dyke intrusions at spreading ridges is relatively short, on the order of a few tens of years. On the other hand, models of melt extraction from a mantle plume produce volcanic pulses on timescales from ≤103 y (Schmeling, 2006), thus affecting the correlated hydrothermal activity above mid plate hot spots. For example, in the case of Hawaii and Iceland, hot spot volcanic activity varies over timescales of 101–103 years (Takada, 1999; Thordarson and Larsen, 2007).

Recently, fields of diffuse venting (Figure 9D) have been observed, for example the Von Damm vent field (between Cuba and South America), located away from the ridge axis, that exhale moderately low pH (6–7) fluids up to 215 C (Hodgkinson, 2015; Lough et al., 2019). Villafañe-Barajas and Colín-Garcia (2021) review submarine hydrothermal vents and their relevance for the origin of life, also emphasizing the widespread nature of diffusive venting.

(García M 2021) In these cases, infiltrating seawater mixes with subsurface hydrothermal fluids, in the process resulting in lower temperature, lower metal content, and less acidic pH fluids compared to the focused flow from chimneys (Lough et al., 2019). Diffuse, seepage of hydrothermal fluids is apparently widespread and makes up 60%–90% of the flux of hydrothermal effluent (German et al., 2016).

Scheirer et al. (2006) noted variations in temperature in both focussed and diffuse hydrothermal vents along the East Pacific Rise on the order of days to weeks. These were controlled by the presence of a shallow, subsurface reservoir of warm hydrothermal fluids that exited and mixed with ambient seawater during eruption/fissuring events at varying rates on a daily to weekly basis. Although this study related only to the temperature of the hydrothermal emissions, it is anticipated that other factors, such as pH and the concentration of dissolved trace metal species would be equally affected.

In terms of spatial scales and hydrothermal vent variability, a recent study of the la Scala vent field in the Woodlark Basin (NE of Australia) by Boulart et al. (2022) documented contemporaneous active and inactive areas, one comprising mainly diffuse vents over an area of 30 × 10 m, and a second area of vigorous black smoker vents in an area of 50 × 15 m. The latter is located above brecciated and altered basaltic rocks. While active venting is occurring in the black smoker area, it was also noted that a previous hydrothermal episode had occurred about 24,000 years ago, i.e. there are repetitive events on time scales of several 104 years.

Interestingly, experimental studies have recently demonstrated the formation mechanisms of the edifices precipitated around black and white smoker type vents. Cardoso and Cartwright (2017) demonstrated that black smoker growth is driven by thermal diffusion, while white smokers, similar to those of the Mid Atlantic Ridge field Lost City, are formed by much slower chemical diffusion. In the latter case, the increased contact between the effluent and the environmental fluids owing to the slower extrusion rate of the effluent and therefore the greater time available for interactions between the ambient seawater, results in fluid dynamics that leads to precipitation, producing a self-organized and self-assembled complex system, and allowing the controlled exchange of ions with the environment across a semipermeable membrane (cf. Russell et al., 1993).

Fields of white smoker vents are more long lasting because they are not directly connected to active volcanic activity and can be active for up to several 105 years. The Lost City white smoker field on the North Atlantic spreading ridge has been dated to more than 120,000 years (Ludwig et al., 2011), and has formed on an extremely slow spreading centre on top of ultramafic basement rocks. Present activity has been ongoing for about 30,000 years. The Lost City site is relatively large, covering about 500 m2, and comprises both active and inactive vents locations.

#### 4.2.2 Shallow water hydrothermal environments

We define shallow water environments as those that were above wave base. This is about 5–15 m for normal conditions today but up to 40 m for storm conditions. During the Hadean/Palaeoarchaean when higher temperatures, more frequent impacts and associated tsunamis (e.g., Lowe et al., 2003), as well as seismic and gravitational slumping would have created more unstable conditions, the storm wave base may have been deeper, perhaps up to 100 m. Shallow water hydrothermal environments during the Hadean/Palaeoarchaean were characterised by the influx of riverine runoff into the shallow basins (cf. Hickman-Lewis et al., 2020b) that formed on top of the plateau-like protocontinents as “aprons” around exposed volcanic edifices. Thus, while the seawater in these basins has the positive Eu signature indicative of hydrothermal fluids that was global during these eras, and while other rare earth element indicators for episodically active, local hydrothermal input were common, the seawater was diluted by riverine input (op.cit.). In such shallow water basins, fresh water and seawater, infiltrating fractures in the basaltic crust, mixed to varying degrees with the subsurface hydrothermal fluids, resulting in an hydrothermal effluent with relatively low temperatures.

We will use the example of one, well-studied, sedimentary basin, the Josefsdal Chert, Barberton Greenstone Belt (3.33 Ga) (Westall et al., 2015a; Westall et al., 2018), to demonstrate hydrothermal activity in a shallow water basin (N.B., this basin is only one example, others exhibit different characteristics). The Josefsdal Chert is a sequence of volcaniclastic, chemical and biogenic sediments deposited on pillow lavas. In general, the sediments comprise three successive layers, during which hydrothermal activity fluctuated in importance depending upon the ambient volcanic setting. The lower layer consisting of volcanic ash fall and detrital volcanic components and was deposited directly on pillow lavas where it was very heavily dissected and infiltrated by hydrothermal silica, both during sedimentation and during early lithification of the sediments. The base of the unit comprises hyaloclastites of brecciated basaltic rock and hydrothermal silica, testifying to the explosive nature of the contact between seawater, hot lava and hydrothermal fluids. This sedimentary unit contains evidence of hydrothermal venting in a littoral, beach setting: here, phototrophic microbial biofilms and mats exposed on the beach surfaces were killed by hydrothermal outflow (as evidenced by the presence of still turgid filaments) and completely impregnated with silica (Westall et al., 2006b; Westall et al., 2011b).

**Figure 10:**
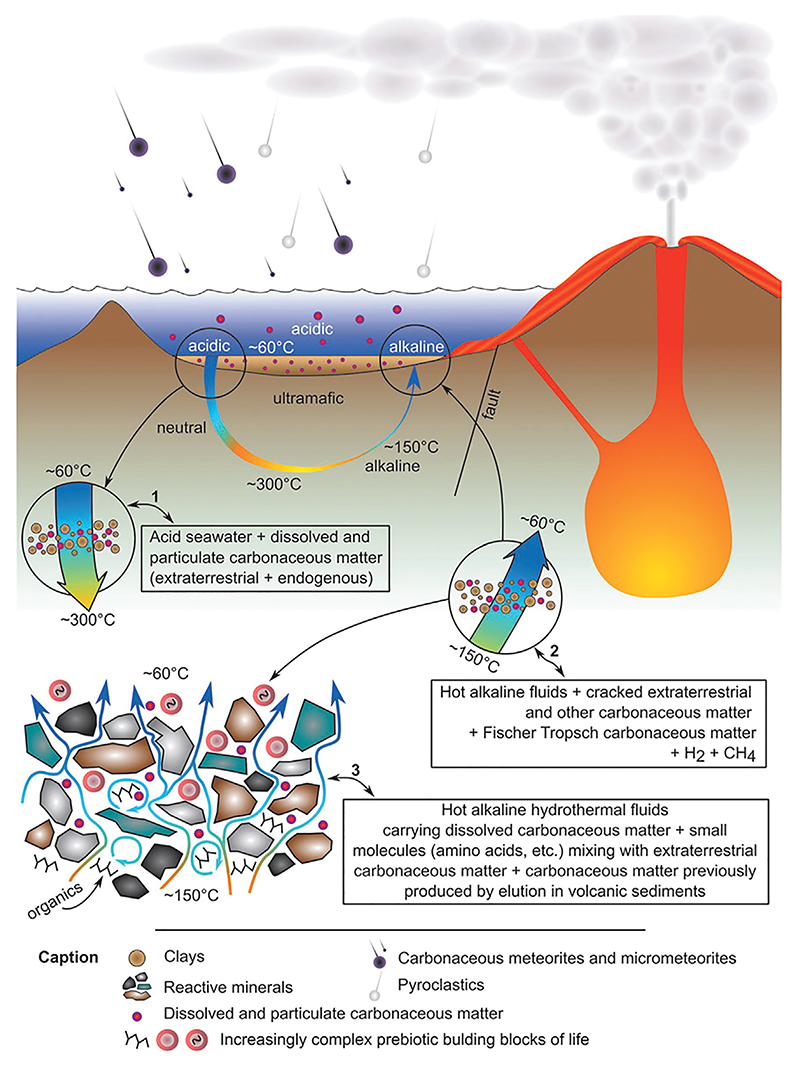
Circulation of hydrothermal fluids through shallow water volcanic sediments on the Hadean/palaeoarchaean Earth (after Westall et al., 2018). This scheme is based on data from well-preserved, Palaeoarchaean rocks of ∼3.5–3.3 Ga. However, the geo-environmental situation of the Palaeoarchaean is considered to be analogous to that of the latter half of the Hadean, when life is believed to have emerged.

Focussed venting took place during the sedimentation of this unit, as evidenced by vein density on the order of 1–2 m (vent features themselves are rare). Diffuse transport of hydrothermal fluids through the accumulating sediment was pervasive, as documented by the almost instantaneous silicification of the sediments, as well as the presence of mini veins and vents of cm size cutting through the sediments (Figures 9, 10). Hydrothermal effluent was also transported through large growth faults that controlled sedimentation thicknesses in many of the Palaeoarchaean basins (de Vries, 2004; de Vries et al., 2010; Westall et al., 2015a; cf Figure 3). Growth faults are normal faults that are active during sedimentation, enabling sediment to accumulate to great thicknesses on the downfault side, while condensed sediment sequences typify the upfault side. Their relevance is that they can be deep reaching and act as important conduits of hydrothermal fluids. Based on comparisons with sedimentation rates in modern volcanic environments, estimations for the duration of sedimentation (and hydrothermal activity) in this first phase, based on the thickness of the sediment layers, is of the order of 3.5–27 cm/ky. For a unit thickness of ∼1 m, this means that sedimentation and contemporaneous hydrothermal activity continued, probably in an episodic manner, over periods of several 103 years. Volcanic and hydrothermal activity gradually waned and were replaced by a long period (105–106 y) of quiescence, during which chemical and biogenic sediments were deposited without being directly influenced by hydrothermal activity (Unit 2). This quiet period was brought to an end by renewed volcanic activity, producing once more point source injections of hydrothermal fluids which would influence the surrounding volcaniclastic sediments for up to 1–2 m (Unit 3) at the base of ashfall into very shallow waters (Unit 4).

Hydrothermal activity (point source and diffuse) was continuous throughout deposition of the basaltic volcanoclastic sedimentary units that could be traced laterally for up to a couple of kilometres (and was probably much more extensive, judging by similar volcanoclastic sedimentary deposits of the 3.472 Ga Middle Marker Chert from Barberton, Lanier and Lowe, 1982).

The type of hydrothermal activity in these shallow water environments appears to have been exclusively low temperature and silica rich (Hofmann and Harris, 2008; Westall et al., 2015a). Primary carbonate deposits, as in the deep sea white smokers of today, were not present, possibly because of the mainly acidic seawater conditions (note that carbonate did form as alteration products of pillow lavas directly exposed to seawater), or because if the relative concentrations of silicon and calcium in the hydrothermal fluids. Secondary carbonate as dolomite, ankerite or siderite were common diagenetic components of the sediments and precipitated contemporaneously with or after the silica gel. These shallow water environments characterised by lower temperature point and diffuse hydrothermal activity may have been suitable locations for the prebiotic chemistry and abiogenesis (Figure 10) (Westall et al., 2018).

High temperature, Fe-rich venting has not been documented in the shallow water environments, although there is one disputed: the iron-rich deposit in the Buck reef Chert (3.42 Ga) in Barberton, that has been described as an ironstone pod, similar to the Fe-rich deposits around deep sea black smokers (de Ronde et al., 1994). This particular deposit has subsequently been re-interpreted as a Quaternary spring, but the debate continues (Lowe and Byerly, 2003).

## 5 Open questions about the origin of life

Even if we have today a more coherent understanding of the environments of the early Earth, still we do not know how life began on our planet. It is generally assumed that life started in a liquid water medium, because water is an ideal solvent (Brack, 1993). Water facilitates the formation of clay minerals *via* aqueous alteration of silicate minerals. Water is also a good heat dissipater, and for example, the products that are synthesized in hot vents could be rapidly quenched in the cooler surroundings thanks to this good heat conductivity. Imai et al. (1999) and Ogata et al. (2000) have demonstrated that oligomers of glycine can be formed under simulated thermal-quenching hydrothermal conditions. Water can also act as a discriminating driver for chemistry, as shown by the polymerization in water of a mixture of amino acids (including proteinaceous amino acids) containing both protein and non-protein amino acids, close to that found in the Murchison meteorite (Brack, 1987). Indeed, one of the most important aspects of water is its electrochemistry power (or redox chemistry), since metabolism can only occur in a polar fluid where electrons are easy to move around.

Theoretical considerations have been proposed for origins of life studies. The field, aiming to bridge chemistry and biology, must include “boundary conditions” as defined by Polanyi (Polanyi 1997) and commented by Paksi (Paksi 2014): structural boundary conditions as found in the field of chemistry and control boundary conditions found in biology. De Duve mentioned “congruence”, the notion that proto metabolism, the set of early chemical reactions that initiated and sustained incipient life, followed pathways that prefigured in many respects those of present-day, enzyme-catalyzed metabolism (de Duve 2003).

However, chemists have to run practical experiments in their laboratories. Even if the primitive environment is now better known, a reasonable scenario for the origin of life still faces the following pending questions:

### 5.1 An organic or mineral start to life?

It is generally assumed that primordial life was based on organic molecules containing carbon and hydrogen atoms associated with oxygen, nitrogen, and sulfur, the basis of current biology. However, some scientists imagined an alternative scenario based on inorganic substrates. Schneider (1977), for example, suggested that the complex dislocation networks found in some crystals could follow the definition of living units, proposing the idea of a crystalline physiology. According to Cairns-Smith (1982), there is no compelling reason to relate the first living cell to a last common ancestor made of organic molecules. He proposed that the first living systems, and the chemical evolution preceding them, could have been based on clay chemistry, different from life as we know it today. Although each step of this hypothetical sequence of events was developed in detail, the scenario has not been subsequently supported by experimental facts. Weiss (1981) published data that appeared to experimentally support Cairns-Smith’s scenario but, unfortunately, the results could not be replicated. Since no satisfactory answers could be obtained, the clay-mediated replication hypothesis will not be further considered here.

### 5.2 Homemade organics from atmospheric gases, from hydrothermal systems or from impact processing?

In his historical experiment, Miller (1953) exposed a mixture of methane, ammonia, hydrogen, and water to spark discharges and silent electric discharge. In his initial experiment, he obtained three amino acids (glycine, alanine, and β-alanine) in the hot water within his experimental flask *via* the intermediary formation of hydrogen cyanide and aldehydes. The synthesis of amino acids occurred efficiently when using a reducing gas mixture containing significant amounts of hydrogen. However, the dominant view now is that the primitive atmosphere consisted mainly of CO_2_, N_2_, and H_2_O, along with small amounts of CO and H_2_ (Catling and Kasting, 2007). Only small yields of amino acids are formed in such a mixture (Schlesinger and Miller, 1983), though more recent studies show that the low yields previously reported could be the outcome of oxidation of the organic compounds during hydrolytic reprocessing by nitrite and nitrate produced in the reactions. The yield of amino acids is greatly increased when oxidation inhibitors, such as ferrous iron, are added prior to hydrolysis, suggesting that synthesis from neutral primitive atmospheres may have been more important than previously thought (Cleaves et al., 2008). Re-analyses of original sample residues from a previously unreported Stanley Miller electric discharge experiment, using a gaseous mixture of H_2_S, CH_4_, NH_3_, and CO_2_, were found to contain a total of 23 amino acids and 4 amines, including 7 organosulfur compounds (Parker et al., 2011). Miller also sparked a gas mixture of CH_4_, NH_3_ and H_2_O while intermittently adding the condensing reagent cyanamide, and modern reanalysis of original sample residues of this experiment found a dozen amino acids, ten glycine-containing dipeptides, and three glycine-containing diketopiperazines (Parker et al., 2014).

The principal driver of organic synthesis is the disequilibrium between very different fluids. Thus, the reducing conditions in hydrothermal systems could have been also an important source of biomolecules on the primitive Earth (Holm and Andersson, 2005; Konn et al., 2015; Sojo et al., 2016; Damer and Deamer, 2020; Rimmer and Shorttle, 2019; White et al., 2020). The reducing environment results from substances dissolved in seawater interacting with inorganic compounds present in very hot crustal material (ultramafic rocks) that reduce them. These reduced compounds flow out of the hydrothermal system and the inorganic sulfides formed precipitate when they mix with cooler ocean water (4°C today but much warmer on the hotter, Hadean Earth, ∼60°C–70°C). For example, hydrocarbons containing 16–29 carbon atoms have been detected in the Rainbow ultramafic hydrothermal system, Mid-Atlantic Ridge (Holm and Charlou, 2001). Hydrothermal vents are often disqualified as efficient reactors for the synthesis of bioorganic molecules, because of their high temperatures, but this applies only to the very hot (>300°C) black smokers, and not vast amount of oceanic crust that provides a three-dimensional substrate through which fluids are flowing, including the cooler (120°C) white smokers and diffuse venting. However, the products that are synthesized in hot vents are rapidly quenched in the surrounding cooler water, which may preserve those organics formed. Recently, hydrothermal-sedimentary environments have been suggested as prebiotic reactors for the origin of life (Westall et al., 2018).

Intense bombardment by meteors and comets probably caused some chemical reprocessing of the Earth’s primitive atmosphere by impact shock chemistry (cf. Maher and Stevenson, 1988; Melosh and Vickery, 1989; Abramov et al., 2013; Mojzsis et al., 2019). This was demonstrated by Furukawa et al. (2009) who subjected a mixture of solid carbon, iron, nickel, water, and nitrogen to high-velocity impacts using a propellant gun. They recovered several organic molecules after the impact, including complex molecules, such as fatty acids and amines. Glycine, the simplest protein-building amino acid, was formed when the starting material contained ammonia, which is believed to have been formed during prior impacts on the early Earth. All the RNA canonical nucleobases and the simplest amino acid glycine were obtained when impacting a mixture of carbon monoxide, methane, and molecular nitrogen using a terawatt high-power laser system in the presence of montmorillonite (Ferus et al., 2020).

**Figure 11:**
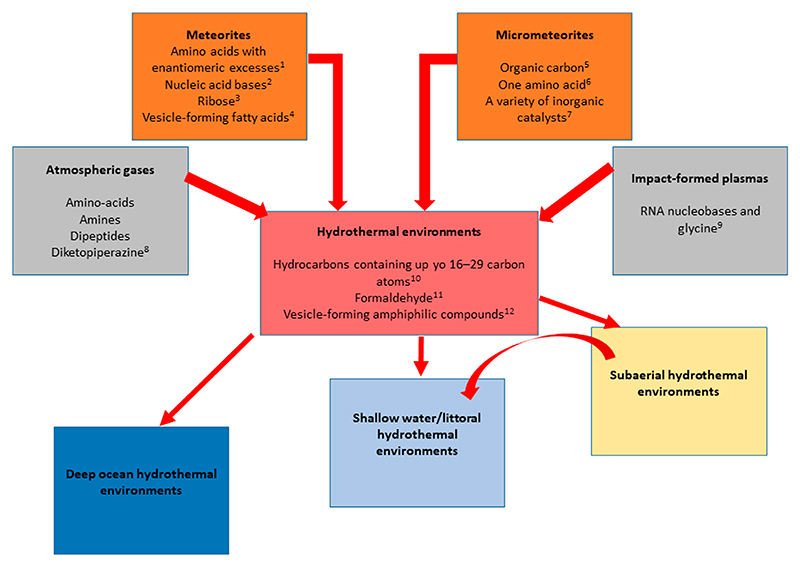
Block diagramme showing the prebiotic products arriving on, or formed on, the early Earth, and illustrating the interactions between different components and different environments. Note that there is no significant transfer of compounds between the subaerial and shallow water hydrothermal environments and those of the deep ocean; any transported molecules into the deep would be so diluted that their impact would be negligible. ^1^Pizzarello et al. (2012), ^2^Stoks and Schwartz (1982), ^3^Furukawa et al. (2019), ^4^Deamer (1998), ^5^Dobrica et al. (2009), Rojas et al. (2021), ^6^Matrajit et al. (2004), ^7^Maurette (2006), ^8^Parker et al. (2014), ^9^Ferus et al. (2020), ^10^Holm and Charlou (2001), ^11^Herschy et al. (2014), ^12^Damer and Deamer (2020).

Figure 11 is a block diagramme showing the prebiotic products arriving on, or formed on, the early Earth, and illustrating the interactions between different components and different environments. Note that there is no significant transfer of compounds between the subaerial and shallow water hydrothermal environments and those of the deep ocean; any transported molecules into the deep would be so diluted that their impact would be negligible. We note here that some of the organic compounds found in the interstellar medium are fairly complex (up to 256 molecular species, Guérlin and Cernicharo, 2022) and may form from radicals made by photolysis (high energy light, up to UV, Öberg, 2016). Some of this material may be the source of meteoritic material (although in the carbonaceous chondrite meteorites this was subsequently reprocessed by aqueous activity); the “kerogen” found in carbonaceous chondrites may be originally of interstellar origin (as shown by the carbon isotopic composition, Swart et al., 1983).

### 5.3 Imported extraterrestrial organics

A great number of organic molecules, including amino acids, have been found in carbonaceous chondrites. The Murchison meteorite, a CM2 type carbonaceous chondrite that fell in Australia in 1969, has been extensively analyzed (Shock and Schulte 1990; Pizzarello, 2007; Pizzarello and Shock, 2010; Pizzarello and Shock, 2017; Glavin et al., 2021). A combination of high-resolution analytical methods applied to the organic fraction of Murchison extracted under mild conditions documented its great chemical diversity of tens of thousands of different molecular compositions and likely millions of diverse structures (Schmitt-Kopplin et al., 2010; Hertkorn et al., 2015). The soluble organic compounds found in the meteorite represent a diverse and abundant group of organics that range from small water-soluble compounds, such as amino acids and polyols, up to 30 carbon-long hydrocarbons.

Vesicle-forming fatty acids have been extracted from different carbonaceous meteorites (Yuen and Kvenvolden, 1973; Deamer, 1985; Deamer, 1998). Nucleic acid bases, purines and pyrimidines, have also been detected in the Murchison meteorite (Stoks and Schwartz, 1982), and ribose was recently identified in the soluble organic matter of carbonaceous chondrites (Furukawa et al., 2019). The total number of meteoritic amino acids detected is now about one hundred, including all the possible α-amino alkylamino acids up to seven carbon atoms, as well as large abundances of N-substituted, cyclic, β, γ, δ, and ε-amino acids, and eight protein-building amino acids. Most of the amino acids detected in carbonaceous chondrites are chiral but are present as racemic mixtures, i.e. L- and D-enantiomers are present in equal proportions. However, Cronin and Pizzarello (1997) found L-enantiomer excesses in six α-methyl-α-amino alkanoic acids from the Murchison (2.8%–9.2%) and Murray (1.0%–6.0%) carbonaceous chondrites, and an enantiomeric excess up to 18% has been measured for isovaline. These amino acids are either unknown or rare in the terrestrial biosphere and cannot therefore be attributed to terrestrial contamination (Pizzarello 2007). In addition, the endogeneity of D- and L-isovaline enantiomers is supported by carbon and hydrogen isotopic data (Pizzarello et al., 2003; Pizzarello and Huang, 2005). Large enantiomeric excesses up to 60% have also been found in Antartica Renazzo-type (CR) chondrites (Pizzarello et al., 2012). The meteoritic enantiomeric excesses figure prominently among the many hypotheses put forward to explain the emergence of homochirality, the one-handedness, of life. Looking for homochiral entities beyond the Earth would probably be one of the best ways to detect extraterrestrial life (Brack, 2019; Gleiser, 2022; Lee et al., 2022).

From dust collections in the Greenland and Antarctica ice sheet (Maurette, 1998; Maurette, 2006), it appears that the Earth captures interplanetary dust as micrometeorites at a rate of about 20,000 tons per year. The value has been lowered to 5,200 tons per year by analyzing recent melts of large volumes of ultra-clean snow at Dome C in Antarctica (Rojas et al., 2021). This value is much higher than the most reliable estimate of the meteorite flux, i.e., about 10 tons per year. At least, ∼20 wt% of the micrometeorites survive unmelted upon atmospheric entry. As their kerogen fraction represents about 2.5 wt% of carbon, this amounts to a total mass of kerogen on the early Earth surface equivalent to a ∼30 m thick global layer (Maurette and Brack, 2006), although this represents an accumulation rate of about 10–6 mm/y over a 500 Ma period. One amino acid, α-amino isobutyric acid, has been identified in Antarctic micrometeorites (Brinton et al., 1998; Matrajt et al., 2004). These grains contain also a high proportion of metallic sulfides, oxides and clay minerals (Folco and Cordier, 2015), a rich variety of inorganic catalysts which could have promoted the reactions of the carbonaceous material which led to the origin of life. Many similarities can be found between Antarctic micrometeorites and the comet Wild 2 samples, in terms of chemical, mineralogical, and isotopic compositions, and in the structure and composition of their carbonaceous matter (Dobrica et al., 2009).

Nevertheless, one of the issues with imported organic matter is what happens to it upon interaction with the early oceans? Certainly carbonaceous chondrites and micrometeorites would have served as raw material sources. However, in the hot, slightly acidic conditions of the early seawaters (especially in shallow water regions where mechanical disruption would have contributed to disaggregation of the exogenic rocks), this material would have been broken down and incorporated into the sedimentary deposits. While numerous layers of Palaeoarchaean extraterrestrial spherule beds are testimony to impacts and the presence of impacted rocks (Lowe et al., 2003; Lowe et al., 2014; Lowe and Byerly, 2015), the recent documentation of finely particulate extraterrestrial organic matter disseminated within early terrestrial sediment (Gourier et al., 2019) is testimony to the fine-scale incorporation of extraterrestrial organic matter into hydrothermal sediments and its availability for reworking, transformation and availability for prebiotic processes.

### 5.4 Primaeval soup or metabolism first for the inception of life?

Two distinct hypotheses have been put forward for the inception of life depending on the availability of organics. In a “metabolism-first” approach, the proponents of an autotrophic life call for the direct formation of simple molecules from carbon dioxide that rapidly evolve to life (Wächtershäuser, 2007). In this scenario, the energy is provided by chemical and thermal disequilibrium. Shock et al. (1995) and McCollom and Shock (1997) note that the geochemical constraints on organic formation and early metabolism are readily met in hydrothermal systems. The energy source required to reduce CO2 could have been provided by the oxidative formation of pyrite from iron sulphide and hydrogen sulphide, giving rise to a two-dimensional mineral surface metabolism. Along with the scenario proposed by Michael Russel (Martin et al., 2008), a laboratory set up simulating conditions prevailing in alkaline hydrothermal vents generated low yields of simple organics (Herschy et al., 2014). So far, the proponents of a “metabolism-first” approach have not been able to produce large enough precursor prebiotic molecules.

In the second hypothesis, the primeval soup scenario, also labelled “replication-first”, preformed complex organic molecules accumulated in a warm little pond, ⟪ à la ⟫ Darwin. Targeting a primitive living cell-like system, great efforts are still being deployed in laboratories to produce boundary molecules providing compartmentalization, protein enzymes and RNA, using either hydrothermal conditions, wet-dry cycling or minerals. Nevertheless, we note that wet-drying cycles are not necessary for dehydration reactions.

For compartmentalization, amphiphilic compounds assemble into membranous vesicles in hydrothermal hot spring water (Monnard and Walde, 2015; Milshteyn et al., 2018; Damer and Deamer, 2020; Deamer et al., 2019). Experiments that demonstrate how different prebiotically-available building blocks can become precursors of vesicle-forming phospholipids have been reviewed elsewhere (Fiore and Strazewski, 2016). Mixtures of C10–C15 single-chain amphiphiles form vesicles in aqueous solutions at temperatures of ∼70 C in the presence of isoprenoids and under strongly alkaline conditions (Jordan et al., 2019). Vesicles functionalized with RNA and peptides could have been an interesting step towards the formation of early protocells (Izgu et al., 2016). The possible origins of a cellular life have been reviewed elsewhere (Schrum et al., 2010).

As for prebiotic peptides, seminal articles include the possible role of short peptides in the steps towards the formation of a protocell (Fishkis, 2007), the formation of peptides on oxide surfaces (Lambert et al., 2013; Kitadai et al., 2017), mineral surface chemistry control for origin of prebiotic peptides (Erastova et al., 2017), ester-mediated amide bond formation driven by wet–dry cycles (Forsythe et al., 2015), or the formation and self-assembly of long prebiotic polypeptides produced by the condensation of non-activated amino acids on oxide surfaces (Martra et al., 2014). Frenkel-Pinter et al. (2020) exhaustively reviewed the present state of the art in this question. More recently, abiotic synthesis and chain extension of unique peptide isomers from free amino acids have been obtained in aqueous microdroplets (Holden et al., 2022). Self-replicating catalytic amyloid peptides have been hypothesized as prebiotic informational and protometabolic entities (Maury, 2018; Rout et al., 2022).

RNA is generally claimed as being the most important molecule for the origin of life (Budin and Szostak, 2010; Bernhardt, 2012; Higgs and Lehman, 2015; Benner et al., 2020). In this respect, significant progress has been made in “one-pot” syntheses. Sutherland’s team, in a relevant example of system chemistry, showed that precursors of ribonucleotides, amino acids and lipids can all be derived by the reductive homologation of hydrogen cyanide and some of its derivatives, and thus that all the cellular subsystems could have arisen simultaneously through common chemistry (Patel et al., 2015). A systems chemistry approach has been recently developed (Strazewski, 2019). The synthesis of pyrimidine nucleosides driven solely by wet-dry cycles has also been reported (Becker et al., 2018). In the presence of phosphate-containing minerals, 5′-mono- and diphosphates also form selectively in one-pot reactions (Becker et al., 2019). Diamidophosphate efficiently phosphorylates a wide variety of potential building blocks, nucleosides/nucleotides, amino acids and lipid precursors, under aqueous conditions. Significantly, higher-order structures, oligonucleotides, peptides and liposomes, are formed under the same phosphorylation reaction conditions (Gibard et al., 2018). Abiotic synthesis of purine and pyrimidine ribonucleosides has been obtained in aqueous microdroplets (Nam et al., 2018). The chemistry of abiotic nucleotide synthesis has been reviewed elsewhere (Yadav et al., 2020).

Despite these encouraging results, it still seems unlikely that life could have started with RNA molecules, because whole RNA strands are not simple enough and yet too difficult to assemble under prebiotic conditions. Our preliminary approximation to answer the question asked is that a promising avenue will perhaps consist in far from equilibrium, open wet-dry cycling organic reactions, occurring on mineral surfaces under hydrothermal-like conditions, and generating a “replication-first” scenario in the primeval soup.

### 5.5 Which time scale for the emergence of life?

How long did it take for life to emerge? We do not know, but the fact that the process had to be driven by a kinetic momentum such that there could be no turning back, no undoing of the haphazard combination of molecules to form a protocell, means that it had to have occurred on shorter time scales rather than longer ones, because of the more unstable surface conditions reigning on the early Earth. However, given the uncertainty of our understanding of conditions on the early Earth, all that we can state is that, not knowing how RNA-based life appeared, it is not possible to estimate the time necessary for life to emerge (Orgel, 1998). This also depends on exactly which steps in the emergence of life are considered in the processes leading to the appearance of an auto-catalytic entity. Do we include the time required for the creation of the prebiotic building bricks of life, or simply the assemblage of the preformed molecules? In the latter case, the process could have started very rapidly or even instantaneously, like solutions that suddenly crystallize upon the addition of a crystal acting as a germ. There is also the possibility that life may have emerged more than once in different places on the planet but could have been extinguished by changing environmental parameters, such as a catastrophic meteoroid or asteroid impact (more common during the Hadean (Marchi et al., 2021), and re-emerged. Could life have emerged simultaneously in numerous locations, where one form was more efficient than the others and took over? These are questions we may not be able to answer on Earth, but perhaps future exploration of Mars, where rocks more than 4 Ga are preserved, may provide some insight into this possibility.

We take, as the context for these considerations, our initial premises, that life emerged after the Moon-forming impact about 4.51 Ga (Barboni et al., 2017) and was well-established and relatively evolved by about 3.5 Ga (Noffke et al., 2013; Hickman-Lewis et al., 2018) if not before (e.g., Hassenkam et al., 2017). Zircon crystals offer strong evidence for crustal water at about 4.19 Ga but habitable conditions conducive to prebiotic chemistry probably existed well before (Sleep et al., 2014). How much time did it take between the emergence of life and advanced life? This would have been a process, not an event, rather than individual, isolated cells, perhaps some kind of symbionts?

### 5.6 What to do next to succeed in creating a simple life form in a test tube?

By demonstrating in 1953 that it was possible to form amino acids—the building blocks of proteins - from methane, Stanley Miller generated the ambitious hope that chemists will be able to create life in a test tube. Despite the tremendous efforts of chemists tackling the problem, the dream has not yet been accomplished. Obviously, experiments should adhere to geologically relevant environments pertinent to the early Earth. A rational methodology involving chemical systems on heterogeneous geological surfaces has been proposed, based on the relationship between prebiotic chemistry and the geological conditions of the Hadean Earth (Dass et al., 2016). A recently-created consortium of researchers seeking to understand the origins of life on Earth and in the Universe will hopefully advance the field (Müller et al., 2022). Novel apparatuses for incorporating natural selection processes have been also proposed (Root-Bernstein and Brown, 2022).

For future research, one approach could be far-from-equilibrium wet-dry cycling (either subaerial exposure or dehydration through chelation to mineral surfaces) of organic reactions occurring repeatedly and iteratively at mineral surfaces under hydrothermal-like conditions, an approach merging geology and chemistry (although we noted above that wet-dry cycling is not essential to concentrating organic molecules). Also, information from biology shows that early life flourished at relatively high temperatures (Woese, 1987; Miller and Lazcano, 1995; Gaucher et al., 2003; Camprubi et al., 2019 and references therein). Slight enantiomeric excesses of the reactants, as well as clays and other minerals, should be used. Concerning clays, they formed as soon as liquid water was present on Earth’s surface through aqueous alteration of the early volcanic rocks. Bernal (1949) listed the advantageous features of clays, i.e., their ordered arrangement, large adsorption capacity, shielding capacity against sunlight, ability to concentrate organic chemicals, and ability to serve as polymerization templates. Given the importance of catalysts for prebiotic chemistry, minerals, such as clays were probably the most efficient partners of the primitive game of life (Brack, 2013; Hansma, 2022; Kloprogge and Hartman 2022) but other minerals with reactive surfaces were present on the early Earth, such as spinels, could have been equally useful (Iqubal et al., 2016). The use of microdroplets represents also an interesting avenue for the synthesis of both peptides and nucleotides (e.g., Wang et al., 2021).

## 6 Concluding reflections

In the first place, constraining the time period in which life emerged on Earth is difficult. We have taken as boundaries for this event the oldest evidence for liquid water at the Earth’s surface (onset of habitability conditions) and the oldest evidence for life.

The oldest evidence of water comes from the oldest zircon crystals having formed by fractionation of melts in the crust that date back to 4.19 Ga (Whitehouse et al., 2017 and references therein), although oxygen and silicon isotope measurements of older zircons (4.4–4.3 Ga) indicate interaction of hydrothermal fluids with crustal materials (Mojzsis et al., 2001; Wilde et al., 2001; Trail et al., 2018). However, there is a debate about the real age of these older zircons (Whitehouse et al., 2017). Modelling suggests that liquid water on Earth’s surface could have existed at habitable temperatures earlier than 4.2 Ga (Sleep, 2010; Sleep et al., 2014). Further constraints on habitability can be obtained by comparative planetology, using Mars as a model to better understand surface processes in the terrestrial planets during the first billion years: the very ancient geological record on Earth has been erased on our very active planet, by plate tectonics, and atmosphere, hydrosphere and biosphere interactions with the surface, and there is a consequent lack of preservation of the Hadean crust. Conversely, on Mars, all major geologic activity came to an end more than 3 Ga ago, and therefore the preservation of the early habitability environmental conditions is better (e.g., Fairén et al., 2010). Decades of robotic exploration have confirmed that early Mars was, indeed, as habitable as early Earth (Domagal-Goldman et al., 2016; McMahon et al., 2018; Moser et al., 2019).

There is suggestive evidence for life on Earth (carbon isotope ratios, hydrocarbon molecules) in highly metamorphosed rocks from the 3.75 Ga Isua terrane, West Greenland (Rosing, 1999; Hassenkam et al., 2017) and possibly in older rocks from 4.1 Ga (carbon isotope rations, Bell et al., 2015). Certainly, by 3.5 Ga life was well established on Earth and had diversified to include chemotrophs as well as phototrophs (e.g., Byerly et al., 1986; Walsh, 1992; Hofmann et al., 1999; Tice and Lowe, 2004; Westall et al., 2015a; Hickman-Lewis and Westall, 2021). We can conclude that life likely emerged after about 4.3 Ga, but certainly before 3.75 Ga, a time uncertainty equivalent to the entire Phanerozoic.

Prebiotic chemistry needs input from biology, geology and geochemistry in order to constrain the conditions of the environments in which life might have emerged on Earth. While large-scale processes, such as formation of emerged landmasses and proto-continents, as well as tectonic regime and fluid flow, formed the backdrop, it is in the local-scale environment that life emerged. These local-scale environments were characterised by mainly mafic and ultramafic rocks, as well as volcanic rocks of more felsic composition. They, in turn, were defined by specific mineralogical associations and geochemical compositions (notably important trace elements), including the alteration products of these protoliths (clays, anatase, other). While each local environment would have been characterised by local conditions in terms of temperature, pH, or ionic concentrations, the gradients essential for driving prebiotic chemistry would have been provided by heterogeneity in the local conditions driven by physical factors (tides, drying cycles, hydrothermal effusions) as well as chemical and other gradients.

One important factor generally neglected in prebiotic chemistry is the significance of hydrothermal silica gel in the early terrestrial aqueous environments. Owing to the more or less ubiquitous hydrothermal activity, silica-rich effluent was common in the early oceans. Added to this was silica from devititrification of the volcanic sediments, silica input *via* rivers, and the concentration of silica in shallow waters by dehydration (as an evaporate mineral). The presence of silica gel could have been an important pathway for the concentration of, and dehydration of, prebiotic molecules. It also places constraints on the rates of processes occurring in submarine (and subaerial spring) environments because dehydration and lithification of the local environment (hydrothermal vents and surrounding sediments) was generally fast.

We note the advantages for the emergence of life that would be provided subaerial environments, such as hot springs located around the edges of collapsed volcanic calderas, where subaerially-exposed surfaces gave access to UV radiation that provided the energy for initiating the prebiotic processes (if it was necessary, and not all share this view). While individual springs may not be long lasting, they occur in swarms that, collectively, can last several tens of thousands of years. We also consider hydrothermal systems (vents and their surrounding sediments), where reactive mineral surfaces were important for prebiotic reactions. Such systems probably existed in the deep ocean (for which there is no crustal preservation), and definitely existed on the shallow water platforms that were the protocontinents, as well as in the littoral beach area. All of these individual environments have individual life times but on different scales. While hydrothermal activity associated with mantle plumes may be ongoing for more than tens of thousands of years, individual springs/vents last for tens of years, but occur in swarms.

On top of these context, considerations still remain that represent fundamental, outstanding questions about the origin of life. In the first place, was the start to life inorganic or organic? The evidence seems both and inorganic and an organic start, metabolism coming from inorganic chemistry but the carbonaceous components clearly from organic sources. While many of the organic molecules used in early prebiotic processes were of extraterrestrial origin, there is growing awareness of the endogenous formation of organic molecules in the environment of the early Earth, in the atmosphere, in the crust, or in the clouds of volcanoes. One of the major controversies concerns the timing of the appearance of early metabolism or of the first replication molecule, or even the bounding membranes. How long did it take for the first cells to emerge? We simply do not know. The answer depends on how an RNA world appeared and on exactly which steps in the emergence of life are considered in the processes leading to the appearance of an auto-catalytic entity. It also depends on the time required for the creation of the prebiotic building bricks of life or simply the assemblage of the pre-formed molecules. In the latter case, the process could have started very rapidly or even instantaneously, like solutions that suddenly crystallize upon the addition of a crystal acting as a trigger.

## Figures and Tables

**Table 1:** Collection of published ages related to core formation, differentiation and crust formation. Ages reported here are both model/intercept ages and absolute ages, but not models of ages (after Brasser et al., 2021).

Earth	Age (Ma)	References
U-Pb age of silicate differentation	4,450–4,500	Manhes et al. (1979), Albarède and Juteau (1984), Allègre et al. (2008)
I-Pu-Xe atmosphere retention age	4,450–4,530	Staudacher and Allègre (1982), Ozima and Podosek (1999), Mukhopadhyay (2012), Avice & Marty (2014), Caracausi et al. (2016)
Hf-W age of core formation	4,450–4,530	Halliday et al. (1996), Yin et al. (2002), Kleine et al. (2002, 2009)
Oldest-known terrestrial zircon	4,380	Valley et al. (2014)
Sm-Nd silicate differentiation age	4,460–4,530	Caro et al. (2003), Boyet & Carlson (2005)
Lu-Hf intercept age of crust formation	4,450–4,500	Harrison et al. (2005, 2008)
**Moon**		
Lu-Hf intercept age of crust formation	4,270–4,510	Taylor et al. (2009), Barboni et al. (2017), Maurice et al. (2020)
Hf-W age of lunar core formation	4,505–4,530	Jacobsen (2005), Kleine et al. (2005), Touboul et al. (2009), Thiemens et al. (2019)

**Table 2:** Elemental compositions of Archaean basalts (tholeites, komatiites, cumulates and evolved tholeites from the Kromberg Formation, Barberton Greenstone Belt, South Africa) from Vennemann and Smith (1999) compared with Mid-Ocean Ridge Basalts from Gale et al. (2013).

	Kromberg Fm. Tholeitic basalt	Komatiites	Cumulates	Evolved tholeites	Gale et al. (2013)
					MORB all
SiO_2_	50,73	48	52,18	66,13	50.17
TiO_2_	1,47	5	0,36	1,45	1.68
Al_2_O_3_	14,75	6,54	3,37	13,63	14.7
Fe_2_O_3_	15,5	12,79	13,29	8,99	
FeO					10.43
MnO	0,2	14	0,27	0,09	0.184
MgO	6,1	22,4	15,09	3,73	7.58
CaO	7.38	9,47	15,02	1,34	11.39
Na_2_O	2,14	0,01	0,36	3,61	2.79
K_2_O	0,8	0	0,01	0,73	0.16
P_2_O_5_	0,17	0,06	0,05	0,3	0.184

**Table 3:** Characteristics of Archaean basalts (Barnes and Arndt, 2019).

Basalt type	Geological setting	Mineralogy	Major elements	Trace elements
Tholeitic	Monotonoous basaltic sequences	Plagioclase and pyroxene	Moderate MgO, TiO_2_, Al_2_O_3_, FeO; Fe enrichment during fractionation	Flat REE, no Nb anomalies
Calc-alkaline	Associated with andesite, dacite, rhyolite	Plagioclase, clinopyroxene+/-oxides	High SiO_2_ and Al_2_O_3_, moderate MgO, low TiO_2_ and FeO; SiO_2_-CaO-alkali enrichment during differentiation	Enriched LREE, negative Nb anomalies
Komatiitic	Associated with komatiite	Olivine and chromite on liquidus, spinifex textures	High MgO, low TiO_2_, Al_2_O_3_, FeO	Al/Ti ratios match associated komatiites

**Table 4:** Compositions of felsic lavas from the 3.46 Ga Duffer Formation and Panorama Formation at Kitty’s Creek (Smithies et al., 2007), compared to recent (36 Ma) rhyolites from Texas (Elliott, 2018). The Palaeaoarchaean rhyolites are enriched in Fe and Mg and are poor in K, compared to the younger rhyolites.

	Duffer formation, ~3,46 Ga	Panorama formation Kitty’s creek, ∼3.46 Ga	Round top rhyolite, Texas, 36 Ma
sample no.	179,726	180,223	262–01
SiO_2_	70,77	74,64	75,5
TiO_2_	0,31	33	0,02
Al_2_O_3_	12,9	15,93	13,31
Fe_2_O_3_	3,73	1,08	1,05
FeO			0,55
MnO	0,06	0	0,07
MgO	0,53	0,83	0,07
CaO	2,12	0,04	0,1
Na_2_O	4,74	0,04	4,82
K_2_O	2,31	5,38	4,28
P_2_O_5_	0,05	0,02	>0,01

## References

[R1] Abramov O, Kring DA, Mojzsis SJ (2013). The impact environment of the Hadean Earth. Geochemistry.

[R2] Adam ZR, Hongo Y, Cleaves HJ, Yi R, Fahrenbach AC, Yoda I (2018). Estimating the capacity for production of formamide by radioactive minerals on the prebiotic Earth. Sci Rep.

[R3] Albarede F, Juteau M (1984). Unscrambling the lead model ages. Geochim Cosmochim Acta.

[R4] Allegr CJ, Manhes G, Göpel C (2008). The major differentiation of the Earth at ∼4.45 Ga. Earth Planet Sci Lett.

[R5] Allwood AC, Rosing MT, Flannery DT, Hurowitz JA, Heirwegh CM (2018). Reassessing evidence of life in 3, 700-million-year-old rocks of Greenland. Nature.

[R6] Allwood AC, Walter MR, Kamber BS, Marshall CP, Burch IW (2006). Stromatolite reef from the early archaean era of Australia. Nature.

[R7] Appel PWU, Fedo CM, Moorbath S, Myers JS (1998). Recognizable primary volcanic and sedimentary features in a low-strain domain of the highly deformed, oldest known (≈ 3.7–3.8 Gyr) Greenstone Belt, Isua, West Greenland. Terra nova.

[R8] Arndt NT, Barnes SJ, Lesher CM (2008). Komatiite.

[R9] Arndt NT, Nisbet EG (2012). Processes on the young Earth and the habitats of early life. Ann Rev Earth Planet Sci.

[R10] Asphaug E, Emsenhuber A, Cambioni S, Gabriel TS, Schwartz SR (2021). Collision chains among the terrestrial planets. III. Formation of the moon. Planet Sci J.

[R11] Avice G, Marty B (2014). The iodine-plutonium-xenon age of the Moon-Earth system revisited. Roy Soc Lond Ser A.

[R12] Bach W, Früh-Green GL (2010). Alteration of the oceanic lithosphere and implications for seafloor processes. Elements.

[R13] Bahcall JN, Pinsonneault MH, Basu S (2001). Solar models: Current epoch and time dependences, neutrinos, and helioseismological properties. Astrophysical J.

[R14] Barboni M, Boehnke P, Keller B, Kohl IE, Schoene B, Young ED (2017). Early formation of the moon 4.51 billion years ago. Sci Adv.

[R15] Barnes SJ, Arndt NT, van Kranendonk MJ (2019). Earth’s oldest rocks.

[R16] Baross JA, Hoffman SE (1985). Submarine hydrothermal vents and associated gradient environments as sites for the origin and evolution of life. Orig Life Evol Biosph.

[R17] Becker S, Feldmann J, Wiedemann S, Okamura H, Schneider C, Iwan K (2019). Unified prebiotically plausible synthesis of pyrimidine and purine RNA ribonucleotides. Science.

[R18] Becker S, Schneider C, Okamura H, Crisp A, Amatov T, Dejmek M (2018). Wet-dry cycles enable the parallel origin of canonical and non-canonical nucleosides by continuous synthesis. Nat Commun.

[R19] Bell EA, Boehnke P, Harrison TM, Mao WL (2015). Potentially biogenic carbon preserved in a 4.1 billion-year-old zircon. Proc Nat Acad Sci.

[R20] Benner SA, Bell EA, Biondi E, Brasser R, Carell T, Kim H-J (2020). When did life likely emerge on Earth in an RNA-first process?. ChemSystemsChem.

[R21] Bernal JD (1949). The physical basis of life. Proc Phys Soc Sect A.

[R22] Bernhardt HS (2012). The RNA world hypothesis: The worst theory of the early evolution of life (except for all the others)a. Biol Direct.

[R23] Bolhar R, Kamber BS, Moorbath S, Whitehouse MJ, Collerson KD (2005). Chemical characterization of Earth’s most ancient clastic metasediments from the Isua greenstone belt, Southern West Greenland. Geochim Cosmochim Acta.

[R24] Boschi C, Früh-Green GL, Delacour A, Karson JA, Kelley DS (2006). Mass transfer and fluid flow during detachment faulting and development of an oceanic core complex, Atlantis Massif (MAR 30 °N). Geochem Geophys Geosyst.

[R25] Boulart C, Rouxel O, Scalabrin C, Le Meur P, Pelleter E, Poitrimol C (2022). Active hydrothermal vents in the Woodlark Basin may act as dispersing centres for hydrothermal fauna. Commun Earth Environ.

[R26] Boyet M, Carlson RW (2005). 142Nd evidence for early (>4.53 Ga) global differentiation of the silicate Earth. Science.

[R27] Brack A, Cavalazzi B, Westall F (2019). Biosignatures for Astrobiology.

[R28] Brack A, Bergaya F, Lagaly G (2013). Handbook of clay science.

[R29] Brack A (1993). Liquid water and the origin of life. Orig Life Evol Biosph.

[R30] Brack A (1987). Selective emergence and survival of early polypeptides in water. Orig Life Evo Biosph.

[R31] Brasie MD, Matthewman R, McMahon S, Wacey D (2011). Pumice as a remarkable substrate for the origin of life. Astrobiology.

[R32] Brasier MD, Matthewman R, McMahon S, Kilburn MR, Wacey D (2013). Pumice from the ∼3460Ma apex basalt, western Australia: A natural laboratory for the early biosphere. Precambrian Res.

[R33] Brasser R, Mojzsis SJ, Werner SC, Abramov O (2021). A new estimate for the age of highly-siderophile element retention in the lunar mantle from late accretion. Icarus.

[R34] Brinton KLF, Engrand C, Glavin DP, Bada JL, Maurette M (1998). A search for extraterrestrial amino acids in carbonaceous Antarctic micrometeorites. Orig Life Evol Biosph.

[R35] Budin I, Szostak JW (2010). Expanding roles for diverse physical phenomena during the origin of life. Annu Rev Biophys.

[R36] Byerly GR, Lower DR, Walsh MM (1986). Stromatolites from the 3, 300–3, 500-myr Swaziland supergroup, Barberton mountain land, South Africa. Nature.

[R37] Cairns-Smith AG (1982). Genetic takeover.

[R38] Cairns-Smith AG (1966). The origin of life and the nature of the primitive gene. J Theor Biol.

[R39] Campbell KA, Guido DM, Gautret P, Foucher F, Ramboz C, Westall F (2015). Geyserite in hot-spring siliceous sinter: Window on earth’s hottest terrestrial (paleo)environment and its extreme life. Earth Sci Rev.

[R40] Camprubí E, De Leeuw JW, House CH, Raulin F, Russell MJ, Spang A (2019). The emergence of life. Space Sci Rev.

[R41] Caracausi A, Avice G, Burnard PG, Füri E, Marty B (2016). Chondritic xenon in the Earth’s mantle. Nature.

[R42] Cardoso SS, Cartwright JH (2017). On the differing growth mechanisms of black-smoker and Lost City-type hydrothermal vents. Proc Roy Soc Lond A.

[R43] Caro G, Bourdon B, Birck JL, Moorbath S (2003). 146Sm-142Nd evidence from Isua metamorphosed sediments for early differentiation of the Earth’s mantle. Nature.

[R44] Catalá TS, Shorte S, Dittma T (2021). Marine dissolved organic matter: A vast and unexplored molecular space. App Microbiol Biotechnol.

[R45] Cates NL, Mojzsis SJ (2007). Pre-3, 750 Ma supracrustal rocks from the Nuvvuagittuq supracrustal belt, northern Québec. Earth Planet Sci Lett.

[R46] Catling D, Kasting JF, Sullivan WT, Baross JA (2007). Planets and life.

[R47] Cavosie AJ, Valley JW, Wilde SA, E.i.m.f (2005). Magmatic δ18O in 4400–3900 ma detrital zircons: A record of the alteration and recycling of crust in the early archean. Earth Planet Sci Lett.

[R48] Cleaves HJ, Chalmers JH, Lazcano A, Miller SL, Bada JL (2008). A reassessment of prebiotic organic synthesis in neutral planetary atmospheres. Orig Life Evol Biosph.

[R49] Cnossen I, Sanz-Forcada J, Favata F, Witasse O, Zegers T, Arnold NF (2007). Habitat of early life: Solar X-ray and UV radiation at Earth’s surface 4–3.5 billion years ago. Geophys Res Planets.

[R50] Connelly JN, Bizzarro M, Krot AN, Nordlund Å, Wielandt D, Ivanova MA (2012). The absolute chronology and thermal processing of solids in the solar protoplanetary disk. Science.

[R51] Connelly JN, Bizzarro M (2016). Lead isotope evidence for a young formation age of the Earth–Moon system. Earth Planet Sci Lett.

[R52] Corliss JB, Baross JA, Hofman SE (1981). An hypothesis concerning the relationship between submarine hot springs and the origin of life on Earth. Oceanol Acta.

[R53] Costerton J, Lewandowski DE, Caldwell DE, Korber DR, Lappin-Scott HM (1995). Microbial biofilms. Ann Rev Microbiol.

[R54] Cronin JR, Pizzarello S (1997). Enantiomeric excesses in meteoritic amino acids. Science.

[R55] Dalrymple GB (2001). The age of the earth in the twentieth century: A problem (mostly) solved. Geol Soc Lond.

[R56] Damer B, Deamer D (2020). The hot spring hypothesis for an origin of life. Astrobiology.

[R57] Dass AV, Hickman-Lewis K, Brack A, Kee TP, Westall F (2016). Stochastic prebiotic chemistry within realistic geological systems. ChemistrySelect.

[R58] Dass AV, Jaber M, Brack A, Foucher F, Kee TP, Georgelin T (2018). Potential role of inorganic confined environments in prebiotic phosphorylation. Life.

[R59] De Duve C (2003). A research proposal on the origin of life. Orig Life Evol Biosphere.

[R60] de Ronde CEJ, de Wit MJ, Spooner ETC (1994). Early Archean (>3.2 Ga) Fe-oxide-rich, hydrothermal discharge vents in the Barberton greenstone belt, South Africa. GSA Bull.

[R61] de Vries ST, Nijman W, de Boer PL (2010). Sedimentary geology of the palaeoarchaean Buck ridge (South Africa) and kittys gap (western Australia) volcano-sedimentary complexes. Precamb Res.

[R62] de Vries ST (2004). Early archaean sedimentary basins: Depositional environment and hydrothermal systems.

[R63] Deamer D, Damer B, Kompanichenko V (2019). Hydrothermal chemistry and the origin of cellular life. Astrobiology.

[R64] Deamer DW, Brack A (1998). Membrane compartments in prebiotic evolution.

[R65] Deamer DW (1985). Boundary structures are formed by organic components of the Murchison carbonaceous chondrite. Nature.

[R66] Dehant V, Debaille V, Dobos V, Gaillard F, Gillmann C, Goderis S (2019). Geoscience for understanding habitability in the solar system and beyond. Space Sci Rev.

[R67] Dittmar T, Koch B, Hertkorn N, Kattner G (2008). A simple and efficient method for the solid-phase extraction of dissolved organic matter (SPE-DOM) from seawater. Limnol Oceanogr Methods.

[R68] Djokic T, Van Kranendonk MJ, Campbell KA, Havig J, Walter MR, Guido DM (2021). A reconstructed subaerial hot spring field in the *3.5 billion-year-old Dresser Formation, North Pole Dome, Pilbara craton, western Australia. Astrobiology.

[R69] Djokic T, Van Kranendonk MJ, Campbell KA, Walter MR, Ward CR (2017). Earliest signs of life on land preserved in ca. 3.5 Ga hot spring deposits. Nat Commun.

[R70] Dobrica E, Engrand C, Duprat J, Gounelle M, Leroux H, Quirico E (2009). Connection between micrometeorites and Wild 2 particles: From antarctic snow to cometary ices. Meteorit Planet Sci.

[R71] Dodd MS, Papineau D, Grenne T, Slack JF, Rittner M, Pirajno F (2017). Evidence for early life in Earth’s oldest hydrothermal vent precipitates. Nature.

[R72] Domagal-Goldman SD, Wright KE, Arney G, Atri D, Azua-Bustos A, Bowman JS (2016). The Astrobiology primer v2.0. Astrobiology.

[R73] Duverger A, Berg JS, Busigny V, Guyot F, Bernard S, Miot J (2020). Mechanisms of pyrite formation promoted by sulfate-reducing bacteria in pure culture. Front Earth Sci.

[R74] Duverger A, Bernard S, Viennet J-C, Miot J, Busigny V (2021). Formation of pyrite spherules from mixtures of biogenic FeS and organic compounds during experimental diagenesis. Geochem, Geophys, Geosyst.

[R75] Elderfield H, Schult A (1996). Mid-ocean ridge hydrothermal fluxes and the chemical composition of the ocean. Ann Rev Earth Planet Sci.

[R76] Elliott BA (2018). Petrogenesis of heavy rare earth element enriched rhyolite: Source and magmatic evolution of the round top laccolith, trans-pecos, Texas. Trans-Pecos, Tex Miner.

[R77] Emsenhuber A, Asphaug E, Cambioni S, Gabriel TS, Schwartz SR (2017). Collision chains among the terrestrial planets. II. an asymmetry between Earth and venusErastova., V, Degiacomi., MT, Fraser., DG, Greenwell HCMineral surface chemistry control for origin of prebiotic peptides. Planet Sci journalNature Commun.

[R78] Erastova V, Degiacomi MT, Fraser DG, Greenwell HC (2017). Mineral surface chemistry control for origin of prebiotic peptides. Nat Commun.

[R79] Fairén AG, Davila AF, Gago-Duport L, Amils R, McKay CP (2009). Stability against freezing of aqueous solutions on early Mars. Nature.

[R80] Fairén AG, Davila AF, Lim D, Bramall N, Bonaccorsi R, Zavaleta J (2010). Astrobiology through the ages of mars: The study of terrestrial analogues to understand the habitability of mars. Astrobiology.

[R81] Fairén AG, Fernández-Remolar D, Dohm JM, Baker VR, Amils R (2004). Inhibition of carbonate synthesis in acidic oceans on early Mars. Nature.

[R82] Fedo C (2000). Setting and origin for problematic rocks from the >3.7 Ga Isua greenstone belt, southern west Greenland: Earth’s oldest coarse clastic sediments. Precamb Res.

[R83] Fegley B, Schaefer L (2013). Treatise on geochemistry.

[R84] Ferris JP, Edelson EH, Mount NM, Sullivan AE (1979). The effect of clays on the oligomerization of HCN. J Mol Evol.

[R85] Ferris JP, Hill AR, Liu R, Orgel LE (1996). Synthesis of long prebiotic oligomers on mineral surfaces. Nature.

[R86] Ferus M, Rimmer P, Cassone G, Knızek A, Civis S, Sponer JE (2020). One-pot hydrogen cyanide-based prebiotic synthesis of canonical nucleobases and glycine initiated by high-velocity impacts on early Earth?. Astrobiology.

[R87] Fiore M, Strazewski P (2016). Prebiotic lipidic amphiphiles and condensing agents on the early Earth. Life.

[R88] Fisher AT, Wheat CG (2010). Seamounts as conduits for massive fluid, heat, and solute fluxes on ridge flanks. Oceanography.

[R89] Fishkis M (2007). Steps towards the formation of a protocell: The possible role of short peptides. Orig Life Evol Biosph.

[R90] Folco L, Cordier C (2015). Micrometeorites. EMU Notes Mineralogy.

[R91] Forsythe JG, Yu S-S, Mamajanov I, Grover MA, Krishnamurthy R, Fernández FM (2015). Ester-mediated amide bond formation driven by wet–dry cycles: A possible path to polypeptides on the prebiotic earth. Angew Chem.

[R92] Foucher F, Westall F, Brandstatter F, Demets R, Parnell J, Cockell CS (2010). Testing the survival of microfossils in artificial martian sedimentary meteorites during entry into earth’s atmosphere: The STONE 6 experiment. Icarus.

[R93] Franck S (1998). Evolution of the global mean heat flow over 4.6 Gyr. Tectonophysics.

[R94] Frenkel-Pinter M, Samanta M, Ashkenasy G, Leman LJ (2020). Prebiotic peptides: Molecular hubs in the origin of life. Chem Rev.

[R95] Furukawa Y, Chikaraishi Y, Ohkouchi N, Ogawa NO, Glavin DP, Dworkin JP (2019). Extraterrestrial ribose and other sugars in primitive meteorites. PNAS.

[R96] Furukawa Y, Sekine T, Oba M, Kakegawa T, Nakazawa H (2009). Biomolecule formation by oceanic impacts on early Earth. Nat Geosci.

[R97] Gagnevin D, Ethien R, Bonin B, Moine B, Féraud G, Gerbe MC (2003). Open-system processes in the Genesis of silica-oversaturated alkaline rocks of the rallier-du-baty peninsula, kerguelen archipelago (Indian ocean. J Volcanol Geotherm Res.

[R98] Gale A, Dalton CA, Langmuir CH, Su Y, Schilling JG (2013). The mean composition of ocean ridge basalts. Geochem Geophys Geosyst.

[R99] Gallo TA, Klein LC (1988). Effect of dehydration on the viscosity of sol-gel processed silica. J Non-Cryst Solids.

[R100] Gaucher EA, Thomson JM, Burgan MF, Benner SA (2003). Inferring the palaeoenviron-ment of ancient bacteria on the basis of resurrected proteins. Nature.

[R101] German CR, Casciotti KA, Dutay JC, Heimbürger LE, Jenkins WJ, Measures CI (2016). Hydrothermal impacts on trace element and isotope ocean biogeochemistry. Phil Trans R Soc A.

[R102] Gibard C, Bhowmik S, Karki M, Kim E-K, Krishnamurthy R (2018). Phosphorylation, oligomerization and self-assembly in water under potential prebiotic conditions. Nat Chem.

[R103] Glavin DP, Elsila JE, Mclain HL, Aponte JC, Parker ET, Dworkin JP (2021). Extraterrestrial amino acids and L-enantiomeric excesses in the CM 2 carbonaceous chondrites Aguas Zarcas and Murchison. Planet Sci.

[R104] Gleiser M (2022). Biological homochirality and the search for extraterrestrial biosignatures. Orig Life Evol Biosph.

[R105] Gourier D, Binet L, Calligaro T, Capelli S, Vezin H, Bréhéret J (2019). Extraterrestrial organic matter preserved in 3.33 Ga sediments from Barberton, South Africa. Geochim Cosmochim Acta.

[R106] Greer J, Caro G, Cates NL, Tropper P, Bleeker W, Kelly NM (2020). Widespread poly-metamorphosed Archean granitoid gneisses and supracrustal enclaves of the Southern Inukjuak domain, Québec (Canada. Lithos.

[R107] Griffin WL, Belousova EA, O’Neill CosyO’Reilly SY, Malkovets V, Pearson NJ (2014). The world turns over: Hadean–Archean crust–mantle evolution. Lithos.

[R108] Guélin M, Cernicharo J (2022). Organic molecules in interstellar space: Latest advances. Front Astron Space Sci.

[R109] Halliday A, Rehkämper M, Lee DC, Yi W (1996). Early evolution of the earth and moon: New constraints from Hf-W isotope geochemistry. Earth Planet Sci Lett.

[R110] Hansell DA, Carlson CA, Repeta DJ, Reiner S (2009). Dissolved organic matter in the ocean: A controversy stimulates new insights. Oceanography.

[R111] Hansma HG (2022). DNA and the origins of life in micaceous clay. Biophys J.

[R112] Harris AC, White NC, McPhie J, Bull SW, Line MA, Skrzeczynski R (2009). Early archean hot springs above epithermal veins, North Pole, western Australia: New insights from fluid inclusion microanalysis. Econ Geol.

[R113] Harrison TM, Blichert-Toft J, Müller W, Albarede F, Holden P, Mojzsis SJ (2005). Heterogeneous hadean hafnium: Evidence of continental crust at 4.4 to 4.5 Ga. Science.

[R114] Harrison TM, Schmitt AK, McCulloch MT, Lovera OM (2008). Early (> 4.5 Ga) formation of terrestrial crust: Lu-Hf, δ18O, and Ti thermometry results for hadean zircons. Earth Planet Sci Lett.

[R115] Hartman H (1975). Speculations on the origin and evolution of metabolism. J Molec Evol.

[R116] Hassenkam T, Andersson M, Dalby K, Mackenzie D, Rosing M (2017). Elements of Eoarchean life trapped in mineral inclusions. Nature.

[R117] Hawkesworth CJ, Cawood PA (2020). The evolution of the continental crust and the onset of plate tectonics. Front Earth Sci.

[R118] Haymon R, White SM, Baker ET, Anderson PG, Macdonald KC, Resing JA (2008). High-resolution surveys along the hot spot–affected galapagos spreading center: 3. Black smoker discoveries and the implications for geological controls on hydrothermal activity. Geochem Geophys Geosyst.

[R119] He R, Hu B, Zhong H, Jin F, Fan J, Hu YH (2019). Reduction of CO2 with H2S in a simulated deep-sea hydrothermal vent system. Chem Commun.

[R120] Herschy B, Whicher A, Camprubi E, Watson C, Dartnell L, Ward J (2014). An origin-of-life reactor to simulate alkaline hydrothermal vents. J Mol Evol.

[R121] Hertkorn N, Benner R, Frommberger M, Schmitt-Kopplin P, Witt M, Kaiser K (2006). Characterization of a major refractory component of marine dissolved organic matter. Geochim Cosmochim Acta.

[R122] Hertkorn N, Harir M, Schmitt-Kopplin P (2015). Nontarget analysis of Murchison soluble organic matter by high-field NMR spectroscopy and FTICR mass spectrometry. Magn Res Chem.

[R123] Hickman-Lewis K, Cavalazzi B, Foucher F, Westall F (2018). Most ancient evidence for life in the Barberton greenstone belt: Microbial mats and biofabrics of the ∼3.47 Ga Middle marker horizon. Precamb Res.

[R124] Hickman-Lewis K, Cavalazzi B, Sorieul S, Gautret P, Foucher F, Whitehouse MJ (2020a). Metallomics in deep time and the influence of ocean chemistry on the metabolic landscapes of Earth’s earliest ecosystems. Sci Rep.

[R125] Hickman-Lewis K, Gourcerol B, Westall F, Manzini D, Cavalazzi B (2020b). Reconstructing Palaeoarchaean microbial biomes flourishing in the presence of emergent landmasses using trace and rare Earth element systematics. Precamb Res.

[R126] Hickman-Lewis K, Westall F (2021). A southern African perspective on the co-evolution of early life and environments. South Afr J Geol.

[R127] Higgs PG, Lehman N (2015). The RNA world: Molecular cooperation at the origins of life. Nat Rev Genet.

[R128] Hodgkinson MRS (2015). The geological controls on the Von Damm vent field.

[R129] Hofmann A, Harris C (2008). Silica alteration zones in the Barberton greenstone belt: A window into subseafloor processes 3.5–3.3 Ga ago. Chel Geol.

[R130] Hofmann HJ, Grey K, Hickman AH, Thorpe R (1999). Origin of 3.45 Ga coniform stromatolites in warrawoona group, western Australia. Geol Soc Am Bull.

[R131] Holden DT, Morato NM, Cooks RG (2022). Aqueous microdroplets enable abiotic synthesis and chain extension of unique peptide isomers from free amino acids. PNAS.

[R132] Holm NG, Andersson EM (2005). Hydrothermal simulation experiments as a tool for studies of the origin of life on Earth and other terrestrial planets: A review. Astrobiology.

[R133] Holm NG, Charlou J-L (2001). Initial indications of abiotic formation of hydrocarbons in the Rainbow ultramafic hydrothermal system, mid-Atlantic ridge. Earth Planet Sci Lett.

[R134] Iler RK (1979). The chemistry of silica.

[R135] Imai EI, Honda H, Hatori K, Brack A, Matsuno K (1999). Elongation of oligopeptides in a simulated submarine hydrothermal system. Science.

[R136] Iqubal MA, Sharma R, Kamaluddin K (2016). Surface interaction of ribonucleic acid constituents with spinel ferrite nanoparticles: A prebiotic chemistry experiment. RSC Adv.

[R137] Izgu EC, Björkbom A, Kamat NP, Lelyveld VS, Zhang W, Jia TZ (2016). N-Carboxyanhydride-Mediated fatty acylation of amino acids and peptides for functionalization of protocell membranes. J Am Chem Soc.

[R138] Jacobsen SB (2005). The Hf-W isotopic system and the origin of the earth and moon. Ann Rev Earth Planet Sci.

[R139] Jerome CA, Kim HJ, Mojzsis SJ, Benner SA, Biondi E (2022). Catalytic synthesis of polyribonucleic acid on prebiotic rock glasses. Astrobiology.

[R140] Johansen A, Ronnet T, Bizzarro M, Schiller M, Lambrechts M, Nordlund A (2021). A pebble accretion model for the formation of the terrestrial planets in the Solar System. Sci Adv.

[R141] Jordan SF, Rammu H, Zheludev IN, Hartley AM, Maréchal A, Lane N (2019). Promotion of protocell self-assembly from mixed amphiphiles at the origin of life. Nat Ecol Evol.

[R142] Karlen D, Price R, Pichler T, Garey J (2010). Changes in benthic macrofauna associated with a shallow-water hydrothermal vent gradient in Papua New Guinea. Pac Sci.

[R143] Kitadai N, Oonishi H, Umemoto K, Usui T, Fukushi K, Nakashima S (2017). Glycine polymerization on oxide minerals. Orig Life Evol Biosph.

[R144] Kleine T, Münker C, Mezger K, Palme H (2002). Rapid accretion and early core formation on asteroids and the terrestrial planets from Hf-W chronometry. Nature.

[R145] Kleine T, Palme H, Mezger K, Halliday AN (2005). Hf-W Chronometry of lunar metals and the age and early differentiation of the Moon. Science.

[R146] Kleine T, Touboul M, Bourdon B, Nimmo F, Mezger K, Palme H (2009). Hf–W chronology of the accretion and early evolution of asteroids and terrestrial planets. Geochim Cosmochim Acta.

[R147] Kloprogge JT, Hartman H (2022). Clays and the origin of life: The experiments. Life.

[R148] Knauth LP (2005). Temperature and salinity history of the Precambrian ocean: Implications for the course of microbial evolution. Palaeogeo Palaeoclimatol Palaeoecol.

[R149] Konn C, Charlou JL, Holm NG, Mousis O (2015). The production of methane, hydrogen, and organic compounds in ultramafic-hosted hydrothermal vents of the Mid-Atlantic Ridge. Astrobiology.

[R150] Korenaga J (2018). Crustal evolution and mantle dynamics through Earth history. Phil Trans Roy Soc A.

[R151] Lagaly G (1984). Clay-organic interactions. Phil Trans R Soc Lond A.

[R152] Lambert J-F, Jaber M, Georgelin T, Stievano L (2013). A comparative study of the catalysis of peptide bond formation by oxide surfaces. Phys Chem Chem Phys.

[R153] Lammer H, Zerkle AL, Gebauer S, Tosi N, Noack L, Scherf M (2018). Origin and evolution of the atmospheres of early venus, earth and mars. Astron Astrophys Rev.

[R154] Lan Z, Kamo SL, Roberts NM, Sano Y, Li XH (2022). A Neoarchean (ca. 2500 ma) age for jaspilite-carbonate bif hosting purported micro-fossils from the Eoarchean (> 3750 ma) Nuvvuagittuq supracrustal belt (Québec, Canada. Precamb Res.

[R155] Lanier WP, Lowe DR (1982). Sedimentology of the Middle marker (3.4 Ga), onverwacht group, transvaal, South Africa. Precamb Res.

[R156] Ledevin M, Arndt N, Chauvel C, Jaillard E, Simionovici A (2019). The sedimentary origin of black and white banded cherts of the Buck Reef, Barberton, South Africa. Geosciences.

[R157] Lee C, Weber JM, Rodriguez LE, Sheppard RY, Barge LM, Berger EL (2022). Chirality in organic and mineral systems: A review of reactivity and alteration processes relevant to prebiotic chemistry and life detection missions. Symmetry.

[R158] Lough AJ, Connelly DP, Homoky WB, Hawkes JA, Chavagnac V, Castillo A (2019). Diffuse hydrothermal venting: A hidden source of iron to the oceans. Front Mar Sci.

[R159] Lourenço DL, Rozel AB, Gerya T, Tackley PJ (2018). Efficient cooling of rocky planets by intrusive magmatism. Nat Geosc.

[R160] Lowe DR (1999). Geologic Evolution of the Barberton Greenstone Belt, South Africa: Geol Soc America Special Paper 29.

[R161] Lowe DR, Byerly GR, Kyte FT, Shukolyukov A, Asaro F, Krull A (2003). Spherule beds 3.47–3.24 billion years old in the Barberton greenstone belt, South Africa: A record of large meteorite impacts and their influence on early crustal and biological evolution. Astrobiology.

[R162] Lowe DR, Byerly G (2003). Ironstone pods in the archean Barberton greenstone belt, South Africa: Earth’s oldest seafloor hydrothermal vents reinterpreted as quaternary subaerial springs. Geology.

[R163] Lowe DR, Byerly GR, Kyte FT (2014). Recently discovered 3.42-3.23 Ga impact layers, Barberton belt, South Africa: 3.8 Ga detrital zircons, archean impact history, and tectonic implications. Geology.

[R164] Lowe DR, Knauth LP (1977). Sedimentology of the Onverwacht Group (3.4 billion years), Transvaal, South Africa, and its bearing on the characteristics and evolution of the early Earth. J Geol.

[R165] Lowe DR, Byerly GR (2015). Geologic record of partial ocean evaporation triggered by giant asteroid impacts, 3.29-3.23 billion years ago. Geology.

[R166] Ludwig KA, Shen C-C, Kelley DS, Cheng H, Edwards RL (2011). U–Th systematics and 230Th ages of carbonate chimneys at the Lost City Hydrothermal Field. Geochim Cosmochim Acta.

[R167] Maher KA, Stevenson DJ (1988). Impact frustration of the origin of life. Nature.

[R168] Manhes G, Allegre CJ, Dupre B, Hamelin B (1979). Lead-lead systematics, the “age of the Earth” and the chemical evolution of our planet in a new representation space. Earth Planet Sci Lett.

[R169] Marchi S, Drabon N, Schulz T, Schaefer L, Nesvorny D, Bottke WF (2021). Delayed and variable late Archaean atmospheric oxidation due to high collision rates on Earth. Nat Geosci.

[R170] Martin W, Baross J, Kelley D, Russell MJ (2008). Hydrothermal vents and the origin of life. Nat Rev Microbio.

[R171] Martra G, Deiana C, Sakhno Y, Barberis I, Fabbiani M, Pazzi M (2014). The formation and self-assembly of long prebiotic oligomers produced by the condensation of unactivated amino acids on oxide surfaces. Angew Chem.

[R172] Matrajt G, Pizzarello S, Taylor S, Brownlee D (2004). Concentration and variability of the AIB amino acid in polar micrometeorites: Implications for the exogenous delivery of amino acids to the primitive Earth. Meteorit Planet Sci.

[R173] Maurette M, Brack A (2006). Cometary petroleum in hadean time? Meteoritics planet. Science.

[R174] Maurette M (1998). Carbonaceous micrometeorites and the origin of life. Orig Life Evol Biosph.

[R175] Maurette M (2006). Micrometeorites and the mysteries of our origins.

[R176] Maurice M, Tosi N, Schwinger S, Breuer D, Kleine T (2020). A long-lived magma ocean on a young Moon. Sci Adv.

[R177] Maury CPJ (2018). Amyloid and the origin of life: Self-replicating catalytic amyloids as prebiotic informational and protometabolic entities. Cell Mol Life Sci.

[R178] McCollom TM (2000). Geochemical constraints on primary productivity in submarine hydrothermal vent plumes. Deep-Sea Res Part I Oceanogr Res Pap.

[R179] McCollom TM, Shock EL (1997). Geochemical constraints on chemolithoautotrophic metabolism by microorganisms in seafloor hydrothermal systems. Geochim Cosmochim Acta.

[R180] McDermott JM, Seewald JS, German CR, Sylva SP (2015). Pathways for abiotic organic synthesis at submarine hydrothermal fields. PNAS.

[R181] McMahon S, Bosak T, Grotzinger JP, Milliken RE, Summons RE, Daye M (2018). A field guide to finding fossils on Mars. J Geophys Res Planets.

[R182] McMahon S (2019). Earth’s earliest and deepest purported fossils may be iron-mineralized chemical gardens. Proc R Soc B Biol Sci.

[R183] Melosh H, Vickery A (1989). Impact erosion of the primordial atmosphere of Mars. Nature.

[R184] Micela G (2002). Evolution of stellar coronal activity on the main sequence. The evolving sun and its influence on planetary environments.

[R185] Micela G, Sciortino S, Serio S, Vaiana GS, Bookbinder J, Golub L (1985). Einstein X-ray survey of the pleiades-the dependence of X-ray emission on stellar age. Astrophysical J.

[R186] Micela G, Sciortino S, Vaiana GS, Schmitt JHMM, Stern RA, Harnden FR (1988). The Einstein observatory survey of stars in the Hyades cluster region. Astrophysical J.

[R187] Miller SL (1953). A production of amino acids under possible primitive earth conditions. Science.

[R188] Miller SL, Lazcano A (1995). The origin of life--did it occur at high temperatures?. J Mol Evol.

[R189] Milshteyn D, Damer B, Havig J, Deamer D (2018). Amphiphilic compounds assemble into membranous vesicles in hydrothermal hot spring water but not in seawater. Life.

[R190] Mojzsis SJ, Brasser R, Kelly NM, Abramov O, Werner SC (2019). Onset of giant planet migration before 4480 million years ago. Astrophys J.

[R191] Mojzsis SJ, Harrison TM, Pidgeon RT (2001). Oxygen-isotope evidence from ancient zircons for liquid water at the Earth’s surface 4, 300 myr ago. Nature.

[R192] Monnard P-A, Walde P (2015). Current ideas about prebiological compartmentalization. Life.

[R193] Morbidelli A, Lunine JI, O’Brien DP, Raymond SN, Walsh KJ (2012). Building terrestrial planets. Ann Rev Earth Planet Sci.

[R194] Moser E, Arcuri GA, Reinhard DA, White LF, Darling JR, Barker IR (2019). Decline of giant impacts on Mars by 4.48 billion years ago and an early opportunity for habitability. Nat Geosc.

[R195] Moyen JF, Martin H (2013). Forty years of TTG research. Lithos.

[R196] Muehlenbachs K, Clayton RN (1976). Oxygen isotope composition of the oceanic crust and its bearing on seawater. J Geophys Res.

[R197] Mukhopadhyay S (2012). Early differentiation and volatile accretion recorded in deep-mantle neon and xenon. Nature.

[R198] Müller UF, Elsila J, Trail D, DasGupta S, Giese C-C, Walton CR (2022). Frontiers in prebiotic chemistry and early Earth environments. Orig Life Evol Biosph.

[R199] Nam I, Nam HG, Zare RN (2018). Abiotic synthesis of purine and pyrimidine ribonucleosides in aqueous microdroplets. PNAS.

[R200] Nijman W, De Vries ST, Eriksson PG, Altermann W, Nelson DR, Mueller WU, Catuneanu O (2004). The precambrian earth: Tempos and events Developments in precambrian geology.

[R201] Nijman W, Kloppenburg A, de Vries ST (2017). Archaean basin margin geology and crustal evolution: An east Pilbara traverse. J Geol Soc.

[R202] Noffke N, Christian D, Wacey D, Hazen RM (2013). Microbially induced sedimentary structures recording an ancient ecosystem in the ca. 3.48 billion-year-old Dresser Formation, Pilbara, Western Australia. Astrobiology.

[R203] Nutman AP, Bennett VC, Friend CR, Van Kranendonk MJ, Chivas AR (2016). Rapid emergence of life shown by discovery of 3, 700-million-year-old microbial structures. Nature.

[R204] O’Neil J, Carlson RW, Paquette J-L, Francis D (2012). formation age and metamorphic history of the Nuvvuagittuq greenstone belt. Precambrian Res.

[R205] O’Neill C, Jellinek AM, Lenardic A (2007). Conditions for the onset of plate tectonics on terrestrial planets and moons. Earth Planet Sci Lett.

[R206] Ogata Y, Imai EI, Honda H, Hatori HK, Matsuno K (2000). Hydrothermal circulation of seawater through hot vents and contribution of interface chemistry to prebiotic synthesis. Orig Life Evol Biosph.

[R207] O’Neil J, Francis D, Carlson RW (2011). Implications of the Nuvvuagittuq greenstone belt for the formation of Earth’s early crust. J Pet.

[R208] Orange F, Westall F, Disnar JR, Prieur D, Bienvenu N, Le Romancer M (2009). Experimental silicification of the extremophilic ArchaeaPyrococcus abyssiandMethanocaldococcus jannaschii: Applications in the search for evidence of life in early earth and extraterrestrial rocks. Geobiology.

[R209] Orcel G, Phalippou J, Hench LL (1986). Structural changes of silica xerogels during low temperature dehydration. J Non-Crystalline Solids.

[R210] Orgel LE (1998). The Origin of Life-How long did it take?. Orig Life Evol Biosph.

[R211] Osinski GR, Cockell CS, Pontefract A, Sapers HM (2020). The role of meteorite impacts in the origin of life. Astrobiology.

[R212] Öberg KI (2016). Photochemistry and astrochemistry: Photochemical pathways to interstellar complex organic molecules. Chem Rev.

[R213] Ozima M, Podosek FA (1999). Formation age of Earth from 129I/127I and 244Pu/238U systematics and the missing Xe. J Geophysl Res.

[R214] Paksi D (2014). The concept of boundary conditions. Polanyiana.

[R215] Papineau D, She Z, Dodd MS, Iacoviello F, Slack JF, Hauri E (2022). Metabolically diverse primordial microbial communities in Earth’s oldest seafloor-hydrothermal jasper. Scie Adv.

[R216] Parker ET, Cleaves HJ, Dworkin JP, Glavin DP, Callahan M, Aubrey A (2011). Primordial synthesis of amines and amino acids in a 1958 Miller H2S-rich spark discharge experiment. PNAS.

[R217] Parker ET, Zhou M, Burton AS, Glavin DP, Dworkin JP, Krishnamurthy R (2014). A plausible simultaneous synthesis of amino acids and simple peptides on the primordial Earth. Angew Chem.

[R218] Pascal R, Pross A, Sutherland JD (2013). Towards an evolutionary theory of the origin of life based on kinetics and thermodynamics. Open Biol.

[R219] Patel BH, Percivalle C, Ritson DJ, Duffy CD, Sutherland JD (2015). Common origins of RNA, protein and lipid precursors in a cyanosulfidic protometabolism. Nat Chem.

[R220] Perchuk AL, Gerya TV, Zakharov VS, Griffin WL (2020). Building cratonic keels in Precambrian plate tectonics. Nature.

[R221] Pizzarello S, Huang Y (2005). The deuterium enrichment of individual amino acids in carbonaceous meteorites: A case for the presolar distribution of biomolecule precursors. Geochim Cosmochim Acta.

[R222] Pizzarello S, Schrader DL, Monroe AA, Lauretta DS (2012). Large enantiomeric excesses in primitive meteorites and the diverse effects of water in cosmochemical evolution. PNAS.

[R223] Pizzarello S, Shock E (2017). Carbonaceous chondrite meteorites: The chronicle of a potential evolutionary path between stars and life. Orig Life Evol Biosph.

[R224] Pizzarello S, Shock E (2010). The organic composition of carbonaceous meteorites: The evolutionary story ahead of biochemistry. Cold Spring Harb Persp Biol.

[R225] Pizzarello S (2007). The chemistry that preceded life’s origin: A study guide from meteorites. Chem Biodivers.

[R226] Pizzarello S, Zolensky M, Turk KA (2003). Nonracemic isovaline in the Murchison meteorite: Chiral distribution and mineral association. Geochim Cosmochim Acta.

[R227] Polanyi M, Allen RT (1997). Michael Polanyi: Society, economics, philosophy Selected papers.

[R228] Polat A, Hofmann AW, Münker C, Regelous M, Appel PWU (2003). Contrasting geochemical patterns in the 3.7–3.8 Ga pillow basalt cores and rims, Isua greenstone belt, southwest Greenland: Implications for postmagmatic alteration processes. Geochim Cosmochim Acta.

[R229] Pollack GH (2001). Cells, gels and the engines of life.

[R230] Raymond SN (2021). A terrestrial convergence. Nat Astron.

[R231] Ricardo A, Carrigan MA, Olcott AN, Benner SA (2004). Borate minerals stabilize ribose. Science.

[R232] Rimmer PB, Shorttle O (2019). Origin of life’s building blocks in Carbon and Nitrogen rich surface hydrothermal vents. Life.

[R233] Robins B, Sandstå NR, Furnes H, de Wit M (2010). Flow banding in basaltic pillow lavas from the early archean hooggenoeg formation, Barberton greenstone belt, South Africa. Bull Volcanol.

[R234] Rogers AD, Brierley A, Croot P, Cunha MR, Danovaro R, Devey C, Larkin KE, Donaldson K, McDonough Ostend N (2015). position paper 22 of the European marine board.

[R235] Rojas J, Duprat J, Engrand C, Dartois E, Delauche L, Godard M (2021). The micrometeorite flux at Dome C (Antarctica), monitoring the accretion of extraterrestrial dust on Earth. Earth Planet Sci Lett.

[R236] Root-Bernstein R, Brown AW (2022). Novel apparatuses for incorporating natural selection processes into origins-of-life experiments to produce adaptively evolving chemical ecosystems. Life.

[R237] Rosing MT (1999). 13 C-depleted carbon microparticles in >3700-Ma sea-floor sedimentary rocks from west Greenland. Science.

[R238] Rossel PE, Stubbins A, Rebling T, Koschinsky A, Hawkes JA, Dittmar T (2017). Thermally altered marine dissolved organic matter in hydrothermal fluids. Org Geochem.

[R239] Rout SK, Rhyner D, Riek R, Greenwald J (2022). Prebiotically plausible autocatalytic peptide amyloids. Chem Eur J.

[R240] Rozel A, Golabek GJ, Jain C, Tackley PJ, Gerya T (2017). Continental crust formation on early Earth controlled by intrusive magmatism. Nature.

[R241] Russell MJ, Daniel RM, Hall AJ (1993). On the emergence of life via catalytic iron-sulphide membranes. Terra nova.

[R242] Russell MJ, Hall AJ (1997). The emergence of life from iron monosulphide bubbles at a submarine hydrothermalredox and pH front. J Geol Soc Lond.

[R243] Russell MJ (2007). The alkaline solution to the emergence of life: Energy, entropy and early evolution. Acta Biotheor.

[R244] Saha A, Yi R, Fahrenbach AC, Wang A, Jia TZ (2022). A physicochemical consideration of prebiotic microenvironments for self-assembly and prebiotic chemistry. Life.

[R245] Sasselov DD, Grotzinger JP, Sutherland JD (2020). The origin of life as a planetary phenomenon. Sci Adv.

[R246] Schaefer L, Fegley B (2010). Chemistry of atmospheres formed during accretion of the Earth and other terrestrial planets. Icarus.

[R247] Scheirer DS, Shank TM, Fornari DJ (2006). Temperature variations at diffuse and focused flow hydrothermal vent sites along the northern East Pacific Rise. Geochem Geophys Geosyst.

[R248] Schlesinger G, Miller SL (1983). Prebiotic synthesis in atmospheres containing CH4, CO, and CO2. J Molec Evol.

[R249] Schmeling H (2006). A model of episodic melt extraction for plumes. J Geophys Res.

[R250] Schmitt-Kopplin P, Gabelica Z, Gougeon RD, Fekete A, Kanawati B, Harir M (2010). High molecular diversity of extraterrestrial organic matter in Murchison meteorite revealed 40 years after its fall. PNAS.

[R251] Schneider J (1977). A model for a non-chemical form of life: Crystalline physiology. Orig Life Evol Biosphere.

[R252] Schreiber U, Locker-Grütjen O, Mayer C (2012). Hypothesis: Origin of life in deep-reaching tectonic faults. Orig Life Evol Biosph.

[R253] Schrum JP, Zhu TF, Szostak JW (2010). The origins of cellular life. Cold Spring Harb Perspect Biol.

[R254] Schubert G, Turcotte DL, Olson P (2001). Mantle convection in the earth and planets 2 volume set.

[R255] Seewald JS, Zolotov MY, McCollom T (2006). Experimental investigation of single carbon compounds under hydrothermal conditions. Geochim Cosmochim Acta.

[R256] Sheilds GA, van Kranendonk MJ, Smithies RH, Bennet VC (2007). Earth’s oldest rocks Dev In precambrian geology.

[R257] Shock EL, Schulte MD (1990). Summary and implications of reported amino acid concentrations in the Murchison meteorite. Geochim Cosmochim Acta.

[R258] Shock EL (1993). Hydrothermal dehydration of aqueous organic compounds. Geochim Cosmochim Acta.

[R259] Shock EL, McCollom T, Schulte MD (1995). Geochemical constraints on chemolithoautotrophic reactions in hydrothermal systems. Orig Life Evol Biosph.

[R260] Shock EL, Schulte MD (1998). Organic synthesis during fluid mixing in hydrothermal systems. J Geophys Res Planets.

[R261] Sim SJ, Stegman DR, Coltice N (2016). Influence of continental growth on mid-ocean ridge depth. Geochem Geophys Geosyst.

[R262] Sizova E, Gerya T, Stüwe K, Brown M (2015). Generation of felsic crust in the archean: A geodynamic modeling perspective. Precamb Res.

[R263] Sleep NH (2016). Asteroid bombardment and the core of Theia as possible sources for the Earth’s late veneer component. Geochem Geophys Geosyst.

[R264] Sleep NH (2010). The hadean-archaean environment. Cold Spring Harb Perspect Biol.

[R265] Sleep NH, Zahnle K, Neuhoff PS (2001). Initiation of clement surface conditions on the earliest Earth. PNAS.

[R266] Sleep NH, Zahnle KJ, Lupu RE (2014). Terrestrial aftermath of the moon-forming impact. Phil Trans Roy Soc A.

[R267] Smithies RH, Champion DC, van Kranendonk MJ, van Kranendonk MJ, Smithies RH, Bennet VC (2007). Earth’s oldest rocksIn Precambrian Geology.

[R268] Sojo V, Herschy B, Whicher A, Camprubi E, Lane N (2016). The origin of life in alkaline hydrothermal vents. Astrobiology.

[R269] Staudacher T, Allegre CJ (1982). Terrestrial xenology. Earth Planet Sci Lett.

[R270] Stein CA, Stein S (1992). A model for the global variation in oceanic depth and heat flow with lithospheric age. Nature.

[R271] Stein CA, Stein S (1994). Constraints on hydrothermal heat flux through the oceanic lithosphere from global heat flow. J Geophys Res.

[R272] Stoks PG, Schwartz AW (1982). Basic nitrogen-heterocyclic compounds in the Murchison meteorite. Geochim Cosmochim Acta.

[R273] Strazewski P (2019). The essence of systems chemistry. Life.

[R274] Swart PK, Grady MM, Pillinger CT, Lewis RS, Anders E (1983). Interstellar carbon in meteorites. Science.

[R275] Takada A (1999). Variations in magma supply and magma partitioning: The role of tectonic settings. J Volcanol Geotherm Res.

[R276] Takahagi W, Seo K, Shibuya T, Takano Y, Fujishima K, Saitoh M (2019). Peptide synthesis under the alkaline hydrothermal conditions on Enceladus. ACS Earth Space Chem.

[R277] Tartèse R, Chaussidon M, Gurenko A, Delarue F, Robert F (2017). Warm Archaean oceans reconstructed from oxygen isotope composition of early-life remnants. Geochem Perspect Lett.

[R278] Taylor DJ, McKeegan KD, Harrison TM (2009). Lu-Hf zircon evidence for rapid lunar differentiation. Earth Planet Sci Lett.

[R279] Thiemens MM, Sprung P, Fonseca ROC, Leitzke FP, Münker C (2019). Early Moon formation inferred from hafnium-tungsten systematics. Nat Geosc.

[R280] Thordarson T, Larsen G (2007). Volcanism in Iceland in historical time: Volcano types, eruption styles and eruptive history. J Geodyn.

[R281] Tice MM, Lowe DR (2004). Photosynthetic microbial mats in the 3, 416-Myr-old ocean. Nature.

[R282] Touboul M, Kleine T, Bourdon B, Palme H, Wieler R (2009). Tungsten isotopes in ferroan anorthosites: Implications for the age of the Moon and lifetime of its magma ocean. Icarus.

[R283] Trail D, Boehnke P, Savage PS, Liu MC, Miller ML, Bindeman I (2018). Origin and significance of si and o isotope heterogeneities in phanerozoic, archean, and hadean zircon. PNAS.

[R284] Trevors JT, Pollack GH (2005). Hypothesis: The origin of life in a hydrogel environment. Prog Biophys Mol Biol.

[R285] Ueda H, Shibuya T, Sawaki Y, Shozugawa K, Makabe A, Takai K (2021). Chemical nature of hydrothermal fluids generated by serpentinization and carbonation of komatiite: Implications for H2-rich hydrothermal system and ocean chemistry in the early Earth. Geochem Geophys Geosyst.

[R286] USGS (2022). Usgs.

[R287] Valley JW, Cavosie AJ, Ushikubo T, Reinhard DA, Lawrence DF, Larson DJ (2014). Hadean age for a post-magma-ocean zircon confirmed by atom-probe tomography. Nat Geosc.

[R288] van den Boorn SH, van Bergen MJ, Nijman W, Vroon PZ (2007). Dual role of seawater and hydrothermal fluids in early archean chert formation: Evidence from silicon isotopes. Geology.

[R289] Van Kranendonk MJ, Baumgartner R, Djokic T, Ota T, Steller L, Garbe U (2021). Elements for the origin of life on land: A deep-time perspective from the Pilbara Craton of Western Australia. Astrobiology.

[R290] Van Kranendonk MJ, Djokic T, Poole G, Tadbiri S, Steller L, Baumgartner R, Van Kranendonk MJ, Bennett V, Hoffmann E (2018). Earth’s oldest rocks.

[R291] Van Kranendonk MJ, Smithies RH, Hickman AH, Champion DC, van Kranendonk MJ, Smithies RH, Bennet VC (2007). Earth’s oldest rocksDev In Precambrian Geology.

[R292] Van Zuilen MA, Lepland A, Teranes J, Finarelli J, Wahlen M, Arrhenius G (2003). Graphite and carbonates in the 3.8 Ga old Isua supracrustal belt, southern West Greenland. Precamb Res.

[R293] Vanderhaeghe O, Guergouz C, Fabre C, Duchêne S, Baratoux D (2019). Secular cooling and crystallization of partially molten Archaean continental crust over1Ga. ComptesRendus Géoscience, Académiedes Sci.

[R294] Vauchez A, Tommasi A, Mainpric D (2012). Faults (shear zones) in the Earth’s mantle. Tectonophysics.

[R295] Vennemann TW, Smith HS, Lowe DL, Byerly GR (1999). Geologic evolution of the Barberton greenstone Belt, South Africa, geological society of America special paper 329.

[R296] Villafañe-Barajas SA, Colín-García MS (2021). Hydrothermal vent systems: the relevance of dynamic systems in chemical evolution and prebiotic chemistry experiments. Int J Astrobiol.

[R297] Wächtershäuser G (2007). On the chemistry and evolution of the pioneer organism. Chem Biodiv.

[R298] Walsh MW (1992). Microfossils and possible microfossils from the early archean onverwacht group, Barberton mountain land, South Africa. Precamb Res.

[R299] Wang W, Qiao L, He J, Ju Y, Yu K, Kan G (2021). Water microdroplets allow spontaneously abiotic production of peptides. J Phys Chem Lett.

[R300] Weiss A (1981). Replication and evolution in inorganic systems. Ang Chem.

[R301] Westall F, Campbell KA, Bréhéret JG, Foucher F, Gautret P, Hubert A (2015b). Archean (3.33 Ga) microbe-sediment systems were diverse and flourished in a hydrothermal context. Geology.

[R302] Westall F, Cavalazzi B, Lemelle L, Marrocchi Y, Rouzaud JN, Simionovici A (2011b). Implications of *in situ* calcification for photosynthesis in a ∼3.3 Ga-old microbial biofilm from the Barberton greenstone belt, South Africa. Earth Planet Sci Lett.

[R303] Westall F, de Ronde CEJ, Southam G, Grassineau N, Colas M, Cockell C (2006b). Implications of a 3.472–3.333 Gyr-old subaerial microbial mat from the Barberton greenstone belt, South Africa for the UV environmental conditions on the early Earth. Phil Trans Roy Soc Lond B.

[R304] Westall F, de Vries ST, Nijman W, Rouchon V, Orberger B, Pearson V, Reimold WU, Gibson R (2006a). Processes on the early earth.

[R305] Westall F, De Wit MJ, Dann J, Van Der Gaast S, De Ronde C, Gerneke D (2001). Early Archaean fossil bacteria and biofilms in hydrothermally influenced, shallow water sediments, Barberton Greenstone Belt, South Africa. Precamb Res.

[R306] Westall F, Foucher F, Bost N, Bertrand M, Loizeau D, Vago JL (2015a). Biosignatures on mars: What, where and how? Implications for the search for martian life. Astrobiology.

[R307] Westall F, Hickman-Lewis K, Hinman N, Gautret P, Campbell KA, Bréhéret JG (2018). A hydrothermal-sedimentary context for the origin of life. Astrobiology.

[R308] Westall F, Höning D, Avice G, Gentry D, Gerya T, Gillmann C (2022). The habitability of venus and a comparison to early Earth. Space Sci Rev.

[R309] Westall F, Steele A, Toporski J, Walsh M, Allen C, Guidry S (2000). Polymeric substances and biofilms as biomarkers in terrestrial materials: Implications for extraterrestrial samples. J Geophys Res Planets.

[R310] Westall F, Foucher F, Cavalazzi B, de Vries ST, Nijman W, Pearson V (2011a). Volcaniclastic habitats for early life on earth and mars: A case study from ∼3.5Ga-old rocks from the Pilbara, Australia. Planet Space Sci.

[R311] Wheat CG, Mottl MJ, Davis EE, Elderfield H (2004). Hydrogeology of the oceanic lithosphere.

[R312] White LM, Shibuya T, Vance SD, Christensen LE, Bhartia R, Kidd R (2020). Simulating serpentinization as it could apply to the emergence of life using the jpl hydrothermal reactor. Astrobiology.

[R313] Whitehouse MJ, Nemchin AA, Pidgeon RT (2017). What can hadean detrital zircon really tell us? A critical evaluation of their geochronology with implications for the interpretation of oxygen and hafnium isotopes. Gondwana Res.

[R314] Wilde SA, Valley JW, Peck WH, Graham CM (2001). Evidence from detrital zircons for the existence of continental crust and oceans on the Earth 4.4 Gyr ago. Nature.

[R315] Woese CR (1987). Bacterial evolution. Microbiol Rev.

[R316] Yadav M, Kumar R, Krishnamurthy R (2020). Chemistry of abiotic nucleotide synthesis. Chem Rev.

[R317] Yin Q, Jacobsen SB, Yamashita K, Blichert-Toft J, Telouk P, Albarede F (2002). A short timescale for terrestrial planet formation from Hf-W chronometry of meteorites. Nature.

[R318] Yuen GU, Kvenvolden KA (1973). Monocarboxylic acids in Murray and Murchison carbonaceous meteorites. Nature.

[R319] Zahnle KJ, Lupu R, Dobrovolskis A, Sleep NH (2015). The tethered moon. Earth Planet Sci Lett.

[R320] Zark M, Christoffers J, Dittmar T (2017). Molecular properties of deep-sea dissolved organic matter are predictable by the central limit theorem: Evidence from tandem FT-ICR-MS. Mar Chem.

[R321] Zawaski MJ, Kelly NM, Orlandini OF, Nichols CI, Allwood AC, Mojzsis SJ (2020). Reappraisal of purported ca. 3.7 Ga stromatolites from the Isua supracrustal belt (West Greenland) from detailed chemical and structural analysis. Earth Planet Sci Lett.

